# A New Demand for Improved Selectivity and Potency of Cyanine Dyes as Antiproliferative Agents Against Colorectal Cancer Cells

**DOI:** 10.3390/molecules29235581

**Published:** 2024-11-26

**Authors:** Ana Maia, Cathy Ventura, Adriana O. Santos, Maria J. Nunes, Renato E. F. Boto, Ângela Sousa, Samuel M. Silvestre, Paulo Almeida, João L. Serrano

**Affiliations:** 1 CICS-UBI—Health Sciences Research Center, University of Beira Interior, Av. Infante D. Henrique, 6201-506 Covilhã, Portugal; anaamaia97@hotmail.com (A.M.); cathy.ventura@ubi.pt (C.V.); asantos@fcsaude.ubi.pt (A.O.S.); rboto@ubi.pt (R.E.F.B.); angela@fcsaude.ubi.pt (Â.S.); sms@ubi.pt (S.M.S.); 2 Department of Chemistry, University of Beira Interior, Rua Marquês de Ávila e Bolama, 6201-001 Covilhã, Portugal; mjnunes@ubi.pt

**Keywords:** asymmetric cyanine dyes, antiproliferative agents, colorectal cancer, cell cycle arrest, topoisomerase II inhibition

## Abstract

Cancer treatment remains a significant challenge, with chemotherapy still being one of the most common therapeutic approaches. Based on our initial studies of symmetric monomethine cyanine dyes, which showed potential against colorectal cancer, this study explored several asymmetric cyanines, aiming to develop more potent and selective antitumor agents, particularly against colorectal cancer. In pursuit of this goal, we have designed, synthesized, and structurally characterized twelve new cyanine dyes. Their antiproliferative effects were then investigated in vitro against both tumor and non-tumor cell lines. Notably, the two most promising dyes in terms of potency and selectivity against Caco-2 colorectal cancer cells were derived from the combination of *N*-methylbenzoxazole and *N*-methylquinoline (dye **5**), as well as *N*-ethylbenzothiazole and *N*-ethyl-6-nitrobenzothiazole (dye **10**). The potential mechanisms behind their antiproliferative action were also explored, revealing that both dyes penetrate cells and localize within the cytoplasm and nucleus. Furthermore, dye **5** was found to slightly induce apoptosis without causing significant cell cycle arrest, in contrast to dye **10**, which increased the number of cells in the G0/G1 phase. Interestingly, both dyes exhibited marked topoisomerase II inhibitory effects, particularly cyanine **5**, which may further explain their antiproliferative activity. Additionally, drug-likeness properties were predicted for both dyes. Overall, cyanine **5** emerged as the most promising candidate for further investigation as a potential treatment for colorectal cancer.

## 1. Introduction

Photodynamic therapy (PDT) is one of the functional applications of cyanine dyes that have been studied extensively. Several comprehensive reviews have been published on this highly versatile class of dyes and their use as photodynamic agents in this relatively recent approach to cancer treatment [1,2,3,4,5,6,7,8,9,10]. In recent years, our research group has made significant contributions to this field, with a particular focus on squarylic derivatives [11].

**Scheme 1:**
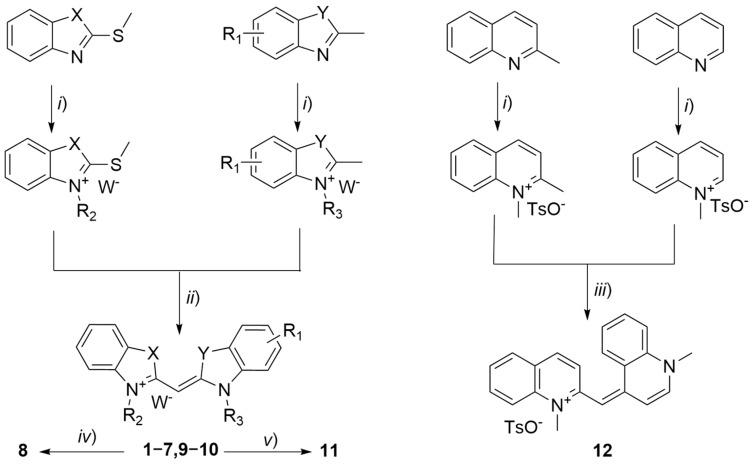
Synthesis of monomethine cyanine dyes **1**–**12**. Conditions: (i) TsOR_2–3_, 120–140 °C or IR_2–3_, acetonitrile, reflux; (ii) GP1 (thioalkyl method via one-pot synthesis: 1° i); 2° pyridine, reflux) or GP3 (thioalkyl method: pyridine or triethylamine/ethanol, reflux) [17]; (iii) GP4 (potassium hydroxide method: KOH, ethanol, reflux) [17]; (iv) **7**, HCl 25%, reflux; (v) **10**, Raney nickel, ethylene glycol, hydrazine hydrate; X and Y are O, S, CH=CH, NCH_3_, or NCH_2_CH_3_; W is I or TsO; R_1_ is H, 5-NHCOCH_3_, (5 or 6)-NH_2_, (5 or 6)-NO_2_; R_2_, and R_3_ are CH_3_, C_2_H_5_, C_5_H_11_, or C_10_H_21_.

**Figure 1:**
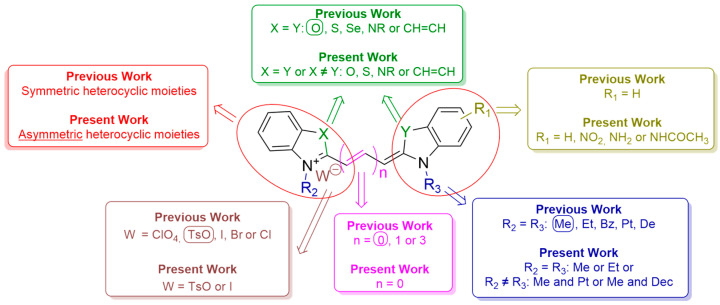
Design of monomethine cyanine dyes studied in the present work, considering the best results from previous work (highlighted with a circle) [17].

While cyanine dyes have shown great potential for several medical applications, including PDT, they still come with dark cytotoxicity which can be concerning but could also offer plausible opportunities for further investigation. In fact, their potential as antitumor agents in the absence of light has been scarcely explored [12,13,14,15,16]. To address this gap, we recently reported the synthesis, structural characterization, and evaluation of the antiproliferative activity of a set of representative symmetrical mono-, tri-, and heptamethine cyanine dyes [17]. A key objective of this published work was to establish structure–activity relationships (SARs) concerning the influence of the heterocycle, counterion, and methine chain length on potency and selectivity. Notably, most of the monomethine and trimethine cyanine dyes examined demonstrated significant antiproliferative effects on human tumor cell lines, including colorectal (Caco-2), breast (MCF-7), and prostate (PC-3) cancer cells. Among these, concentration-viability curves revealed higher potency and selectivity against the Caco-2 cell line. A monomethine cyanine dye derived from benzoxazole emerged as the most promising compound, with an IC_50_ of 0.67 µM for Caco-2 and a selectivity index (SI) of 20.9 compared to normal human dermal fibroblasts (NHDF). Considering the crucial conclusions of this study, particularly the observed residual photodegradation, the absence of altered cytotoxic effects under light exposure, the remarkable cytotoxicity against colorectal cancer cells, and the high SI, it becomes evident that cyanine dyes hold great potential as antiproliferative agents. This discovery has driven us to further develop other cyanine dyes with enhanced cytotoxicity and selectivity, particularly against colorectal cancer. For this, the primary distinction of the present study (Figure 1), compared to previously reported cyanine dyes [17], in addition to the deliberate use of monomethine cyanine dyes optimized for the outer range of the “phototherapeutic window” (650–850 nm), is their intentional asymmetrical design. This asymmetry was achieved by selecting specific substituents at R_2_ and R_3_ positions (**1**–**2**), modifying the heterocyclic ring structure (**3**–**6**, **9**, **12**), and/or introducing nitrogen-substituted R_1_ on one of the terminal heterocyclic rings (**7**–**11**) (Scheme 1).

Herein we report the synthesis and thorough structural characterization of twelve symmetrically substituted monomethine cyanine dyes. In addition, in vitro evaluations are described as methods for evaluating antiproliferative effects on human cell lines, and the IC_50_ values were determined, with the SI calculated. This assessment also includes an evaluation of their activity on topoisomerase II, a microscopic investigation of cellular morphology, and a flow cytometric analysis of apoptosis and cell cycle impacts in Caco-2 cancer cells, as well as in silico-predicted physiochemical and drug-likeness properties.

## 2. Results and Discussion

### 2.1. Synthesis and Structural Characterisation

Asymmetric monomethine cyanine dyes **1**–**7**, **9**–**10**, and **12**, featuring benzothiazole, benzoxazole, benzimidazole, and quinoline as terminal nitrogen heterocycles, were successfully synthesized using established methodologies. These methods include the thioalkyl method via one-pot synthesis (GP1), the thioalkyl method (GP2), and the potassium hydroxide method (GP3) [17]. Aminocyanines **8** and **11** were derived from acetamidocyanine **7** through acid hydrolysis and from nitrocyanine **10** through reduction, respectively (Scheme 1). The asymmetry of these dyes is due to the nature of the heterocycle and/or the chains attached to their terminal nitrogen (**1**–**6**, **9**, **12**), as well as the presence of substituents on one of the heterocyclic rings (**7**–**11**). Finally, considering the results of our previous work [17], the counterions selected (tosylate and iodide) were chosen for their ability to enhance potency and selectivity.

The proton (^1^H) and carbon (^13^C) nuclear magnetic resonance (NMR) spectroscopic characterization of all cyanine dyes **1**–**12** is consistent with their expected structures. To the best of our knowledge, monomethine cyanines **1**–**2** and **7**–**10** have not been previously reported; therefore, high-resolution mass spectrometry (HRMS) data were also acquired for these dyes. Additionally, the novelty of cyanines **4**–**6** and **12** lies in the nature of their counter-ions. Furthermore, cyanines **3** and **11**, while previously described [18,19], show notable differences in physical properties: cyanine **3** exhibits a melting point (m.p.) nearly 30 °C higher than previously reported, and the m.p. for cyanine **11** is not found in the literature.

Characteristic meso methine and N^1/2+^CH_n_ ^1^H and ^13^C NMR signals are detailed in Table S1. The ^1^H and ^13^C NMR spectra further verify the identity of the methine dyes, demonstrating a singlet ranging from 5.62 to 6.85 ppm in the ^1^H NMR spectra (and from 67.6 to 94.4 ppm in the ^13^C NMR spectra) across monomethine cyanine dyes **1**–**12**. The asymmetrical nature of these cyanine dyes is distinctly highlighted by the presence of two typical unshielded protons adjacent to the carbon of the ammonium atom, exhibiting signals between 3.70 to 4.77 ppm in the ^1^H NMR spectra (and between 30.6 and 46.2 ppm in the ^13^C NMR), depending on the nature of the terminal nitrogen heterocycles.

Monomethine cyanine dyes **1**–**11** exhibit maximum absorption wavelengths ranging from 385 to 483 nm, which falls outside the ‘phototherapeutic window’ of 650–850 nm, as intentionally designed (Table 1). Cyanine **12** is herein considered a monomethine dye due to the presence of a single methine group linking the two terminal heterocycles. Therefore, the higher λ_max_ of 558 nm observed for this cyanine dye can be attributed to the extension of five conjugated carbons between the two terminal nitrogens in the 2- and 4-substituted quinolines.

### 2.2. In Vitro Studies

#### 2.2.1. Antiproliferative Effects in Human Cell Lines

To evaluate the anticancer potential of monomethine cyanine dyes **1**–**12**, a cell proliferation study was conducted using the 3-(4,5-dimethylthiazol-2-yl)-2,5-diphenyltetrazolium bromide (MTT) assay. For this preliminary evaluation, a concentration of 10 µM for each dye was tested, with the well-established chemotherapy agent 5-fluorouracil (5-FU) serving as a positive control. The assay included normal human dermal fibroblasts (NHDF) as non-tumor cells, alongside tumor cell lines from colorectal (Caco-2), prostate (PC-3), and breast (MCF-7) adenocarcinomas.

**Figure 2:**
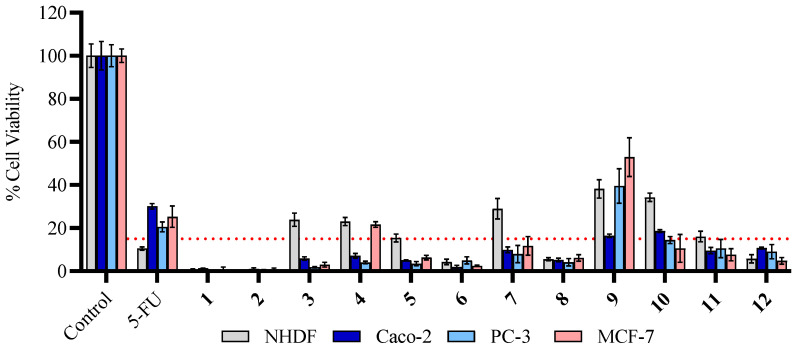
In vitro effect of cyanine dyes **1**–**12** and 5-fluorouracil (5-FU) on the cell viability of normal human dermal fibroblasts (NHDF), and human adenocarcinoma cell lines of the colorectal (Caco-2), prostate (PC-3), and breast (MCF-7) cells, at the single concentration of 10 µM and after 72 h of incubation. Results are expressed as average values ± standard deviation of at least two independent assays performed in quadruplicate. A *p*-value of <0.001 compared to the negative control was observed for all compounds in the statistical significance analysis (Student’s *t*-test).

Upon examining the screening results (Figure 2), it is clear that cyanine dyes **1**–**12** led to a significant reduction in cell viability percentages across all tested cell lines at a concentration of 10 µM. Moreover, a more pronounced cytotoxic effect was noted for most compounds in the tumor cell lines compared to the normal human dermal fibroblasts (NHDF), as evidenced by their selectivity for cancer cells. This selectivity is superior to that observed for the symmetric cyanine dyes previously reported, highlighting the potential therapeutic advantages of these asymmetric cyanine dyes [17]. This phenomenon was prominently evident in the results for dyes **3**, **5**, **7**, and **10**, which led to marked cytotoxic effects across all studied cancer cell lines. Additionally, cyanine **4** originated relevant antiproliferative effects in Caco-2 and PC-3 cell lines but showed reduced efficacy in the MCF-7 line. Similarly, dye **9** had decreased effectiveness in the PC-3 and MCF-7 cells compared to its impact on the Caco-2 cells. Remarkably, except for dye **9**, all the dyes under investigation displayed higher cytotoxicity than the clinically used anticancer drug 5-FU.

Considering the preliminary screening results at a concentration of 10 µM, and based on previously reported data for symmetrical dyes [17], a set of these cyanines under study was selected for further concentration-response studies, including the determination of IC_50_ values. Priority was given to dyes that exhibited less than 15% cell viability in cancer cell lines while exceeding this threshold in the non-cancer NHDF cell line. Consequently, dyes **3**–**5**, **7**, and **10**–**11** were chosen for evaluation on PC-3 cells, and dyes **3**, **5**, **7**, and **10**–**11** were evaluated on MCF-7 cells. Due to their promising potency, and the SI results for the Caco-2 cell line (Table 2), dyes **3**–**5**, **7**, and **9**–**11** were further studied in these cells. Additionally, these dyes were tested in NHDF cells to determine their SI for cancer versus non-cancer cells. The effects of the reference drug 5-FU were evaluated across all cell lines to establish a benchmark for comparison.

A thorough examination of Table 2 provides insights into relevant structure-activity relationship (SAR) data. Previous studies have shown that the antiproliferative potency of symmetric cyanine dyes on Caco-2 cells decreases as the length of the alkyl groups (R_2_ = R_3_) increases. Furthermore, this increase in chain length also reduces selectivity between Caco-2 and NHDF cells [17]. In this context, dyes **1** (IC_50_ = 0.36 µM; SI = 1.5) and **2** (IC_50_ = 0.24 µM; SI = 1.1) also feature asymmetric substituent groups at R_2_ (methyl) and R_3_ (pentyl for **1** and decyl for **2**). Interestingly, there was a slight increase in potency for Caco-2 cells with a longer chain length, contrary to expectations. However, the SI decreased with chain elongation, which aligns with previous findings [17].

To investigate the impact of different heterocyclic ring types and whether the asymmetry is relevant for the antiproliferative efficacy of monomethinocyanines (where X ≠ Y), we tested various rings, including benzothiazoles, benzoxazoles, benzimidazoles, and quinolines. For dyes **3**–**6**, which vary in heterocycle type, some demonstrated notably high SIs for tumor cells. This significant antiproliferative potential suggests that the type of heterocyclic ring plays a crucial role in the cytotoxic properties of these dyes.

The conjugation of benzothiazole with benzoxazole in cyanine **3** (Caco-2 IC_50_ = 0.09 µM; SI = 81.2) and with benzimidazole in dye 4 (Caco-2 IC_50_ = 0.23 µM; SI = 33.6) suggests that introducing asymmetry in the heterocyclic components can significantly enhance the antiproliferative effects of cyanine dyes. Notably, dye **3** not only exhibited potent effects on colorectal cancer cells but also proved to be the most effective dye among those studied against breast cancer cells (MCF-7 IC_50_ = 0.43 µM; SI = 17.2). The combination of quinoline with benzoxazole in cyanine **5** (Caco-2 IC_50_ = 0.01 µM; SI = 278.3) further highlights the promising impact of heterocyclic asymmetry. Dye **5** emerged as the most potent dye in this set of monomethinocyanines for the Caco-2 cell line with remarkable selectivity for these cells compared to the other two cancer cell lines studied. Consistent with previous findings for symmetric cyanines [17], conjugation involving the benzoxazole heterocycle not only enhanced potency for Caco-2 cells but also led to higher selectivity. Finally, combining quinoline with benzothiazole in cyanine **6** (Caco-2 IC_50_ = 0.27 µM; SI = 2.9) or with quinoline (2,4) in dye **12** (Caco-2 IC_50_ = 0.73 µM; SI = 0.9) resulted in a marked reduction in both potency and selectivity, as shown in the screening results (Figure 1). Previous studies with quinoline-derived symmetric monomethine cyanine dyes (2,2 or 4,4) also demonstrated weak SIs for Caco-2 cells compared to non-tumor NHDF cells [17].

The presence and position of substituent groups on the heterocyclic rings play an important role in the selectivity and cytotoxicity of cyanine dyes. To explore this, dyes **7–11** were tested. Among them, cyanine **7** features an amide group at position 5 of the benzothiazole ring, while dye **8** has an amino group at the same position. In contrast, dye **11** has an amino substituent at position 6, whereas dye **10** presents a nitro group at this position. Comparing dyes **8** and **11** reveals that the position of the substituent on the benzene ring significantly impacts antiproliferative effects. Specifically, cyanine **11** (Caco-2 IC_50_ = 0.17 µM; SI = 17.1) is notably more potent and selective for cancer cell lines than cyanine **8** (Caco-2 IC_50_ = 0.74 µM; SI = 1.3). Comparing dyes **11** and **10**, which share the same substituent position but differ in functional groups (amino for dye **11** and nitro for dye **10**), it becomes evident that the nitro group enhances selectivity (SI = 151.4 vs. 17.1), while the antiproliferative potential remains relatively similar (IC_50_ = 0.17 vs. 0.14 µM). A similar trend is seen in cyanines **7** and **8**, which are substituted at the same ring position with amide and amino groups, respectively. Interestingly, the amide group in cyanine 7 significantly enhances both potency (IC_50_ = 0.14 µM vs. 0.74 µM) and selectivity (SI = 80.5 vs. 1.3).

These results underscore the variability in effects among certain cyanine dyes, such as **3**, **5**, **7**, and **10**–**11**, depending on the cancer cell line studied. Some dyes not only demonstrated selectivity between cancer and non-cancer cells but also showed enhanced efficacy in specific tumor cell lines. For instance, dye **3** exhibited notable activity in the PC-3 cell line (IC_50_ = 0.48 µM; SI = 17.2) while dyes **7** (0.38 µM; SI = 30.2) and **11** (0.33 µM; SI = 9.0) showed promising results in the MCF-7 cell line. However, while no single dye emerged as a leading candidate for both PC-3 or MCF-7 cells, dyes **5** (SI = 278.3) and **10** (SI = 151.4) displayed the highest selectivity for Caco-2 cells, although their antiproliferative activity was lower in the other cell lines tested. In terms of the SAR against Caco-2 cells, it is noteworthy that the SI decreased with increasing alkyl chain length and that asymmetry in the heterocyclic components significantly enhanced antiproliferative effects, as seen with the combination of quinoline and benzoxazole rings in cyanine **5**. Additionally, both the presence and position of substituent groups on the heterocyclic rings influenced selectivity and cytotoxicity, with particular emphasis on the nitro group at position 5 of the benzothiazole ring in cyanine **10**.

Taking in mind this SAR analysis, the two most potent and selective cyanines, **5** and **10**, were selected for further investigation. These studies included the evaluation of photocytotoxicity and photostability, analysis of cell morphology and internalization, assessment of apoptosis and/or cell cycle arrest induction, topoisomerase II inhibition, and in silico drug-likeness predictions.

While this work focused on monomethine cyanine dyes designed to be used at the outer edge of the “phototherapeutic window” (650–850 nm), photocytotoxicity and photostability studies were carried out for cyanines **5** and **10**, as part of an effort to determine whether there would be synergistic action from light exposure on their cytotoxicity. A 30 W RGB LED light source, previously validated by our research group [17], was applied to the NHDF cell line using the MTT assay. The results showed that after 30 min of white light irradiation, the cytotoxicity of dye **5** decreased, with the IC_50_ shifting from 2.79 to 4.07 µM. Similarly, for cyanine dye **10**, the IC_50_ increased from 22.38 to 34.06 µM. These reductions in cytotoxicity align with previous studies on similar compounds and are likely due to some level of photodegradation [17].

**Figure 3:**
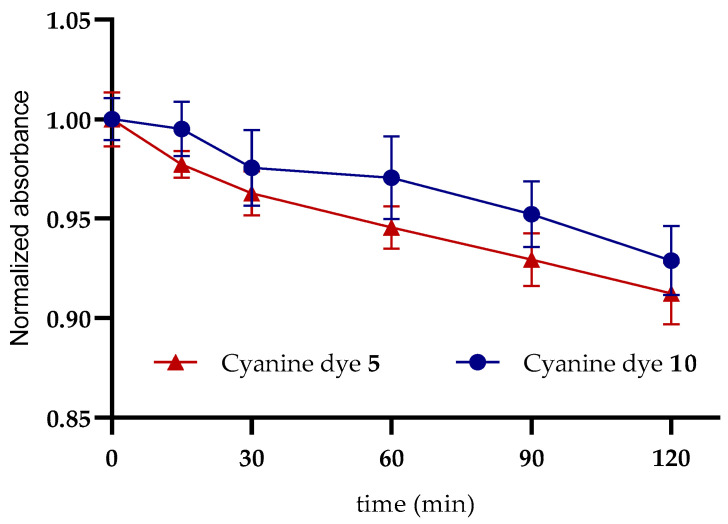
Photostability evaluation of dyes **5** and **10**. Results represent absorbance decay at the maximum absorption wavelength of each dye under study (436 nm for **5** and 439 nm for **10**) as a function of the irradiation time. Absorbance was normalized and presented as arbitrary units (a.u.). The results are expressed as the mean ± standard deviation of two independent assays performed in quadruplicate.

To further explore this, the photostability of these dyes was tested. As shown in Figure 3, a slight degradation was observed, indicated by a reduction in absorbance at the maximum wavelength of each dye after light irradiation. After 30 min of irradiation, the duration used for the photocytotoxicity assay, a 3.7% and 2.5% reduction in absorbance was observed for cyanine dyes **5** and **10**, respectively. A gradual linear degradation continued for at least 120 min. This degradation likely explains the decreased cytotoxicity observed in the photocytotoxicity assay. However, we do not view this as a negative outcome, as it confirms the absence of any photodynamic activity in cyanine dyes **5** and **10**.

#### 2.2.2. Caco-2 Morphological Analysis

**Figure 4:**
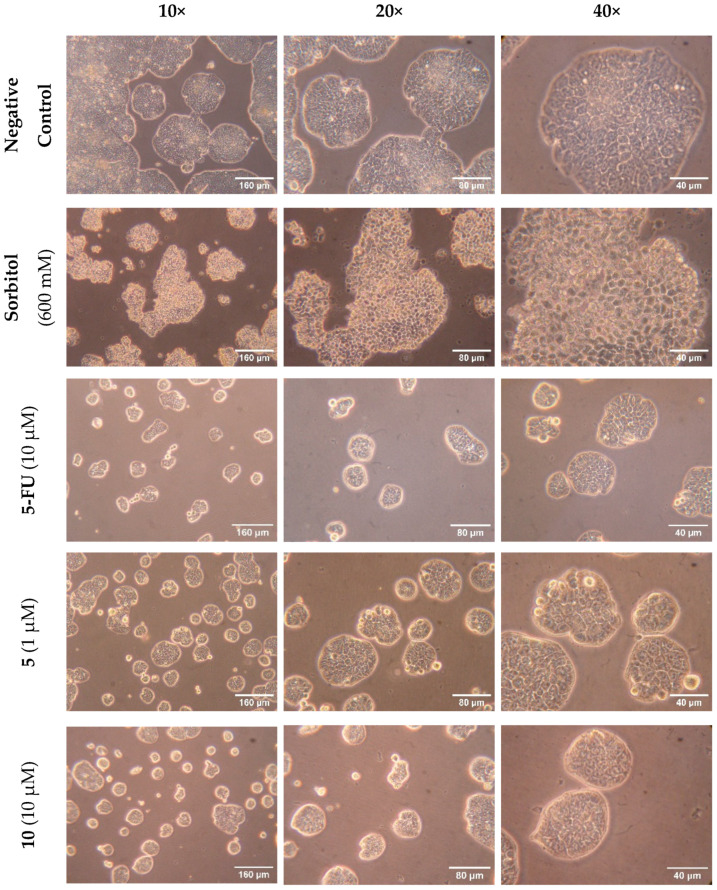
Microscopic images of Caco-2 cells treated 5-FU (10 µM), sorbitol (600 mM), and with cyanine dyes **5** (1 µM) and **10** (10 µM). Images were recorded at 10, 20, and 40× magnification after 72 h of incubation with 5-FU and dyes **5** and **10**. Sorbitol was incubated for 24 h.

Microscopy images of Caco-2 cells were analyzed to evaluate the effects of the most promising dyes, **5** and **10**, on the morphological characteristics of this cell line. These observations were conducted at three different magnifications of 10×, 20×, and 40× as shown in Figure 4. Sorbitol and 5-FU were used as positive controls, while untreated cells served as the negative control.

At a lower magnification of 10×, a significant reduction in cell growth was observed in Caco-2 cells treated with 5-FU and cyanine dyes **5** and **10**. As the magnification increased to 20× and 40×, specific morphological changes became more apparent. In cells treated with 10 µM 5-FU for 72 h, enlarged cells and apoptotic bodies were noted compared to untreated cells, suggesting a potential S phase arrest and some apoptosis induction, consistent with the flow cytometry results. In contrast, treatment with 600 mM sorbitol for 24 h led to a marked increase in apoptotic bodies, indicating that apoptosis may be the primary mechanism of action.

Caco-2 cells treated with cyanine dyes **5** and **10** for 72 h, at concentrations of 1 µM and 10 µM, respectively, exhibited morphological similarities to those treated with 5-FU and were distinct from untreated cells or those treated with sorbitol. A reduction in cell growth, along with the presence of smaller colonies, was observed compared to untreated cells. Treatment with dye **5** also resulted in a few apoptotic bodies and cells with dimensions and morphology similar to those of the negative control. In contrast, dye **10** appeared to induce a noticeable reduction in the size of Caco-2 cells. Additionally, the absence of refringent cells in mitosis suggests a potential arrest in the G0 or G1 phase of the cell cycle.

#### 2.2.3. Fluorescence Microscopy

**Figure 5:**
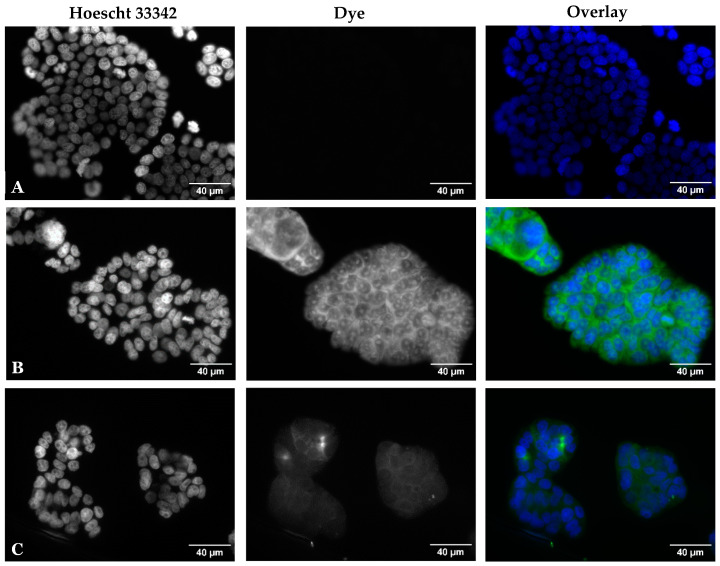
Representative fluorescence microscopy images of the Caco-2 cell line incubated with cyanine dye **5** (**B**) and cyanine dye **10** (**C**) at a concentration of 1 μM for 20 h (both show a signal in the green channel). Control (**A**) was marked only with Hoescht 33342 (blue channel). Images obtained at 40× magnification.

Given that cyanine dyes typically exhibit fluorescence upon appropriate excitation, fluorescence microscopy was performed to determine whether dyes **5** and **10**, at a concentration of 1 µM, could penetrate the cell membranes and to assess their potential intracellular localization in Caco-2 cells (Figure 5). The cells were incubated with the dyes for 20 h, followed by fixation and staining with Hoescht 33342, a commonly used cell-permeant nucleic acid stain.

The results indicate that both tested cyanine dyes exhibit fluorescence and are capable of crossing the cell membrane. Regarding intracellular localization, the dyes label both the cytoplasm (with diffuse and heightened intensity) and the nucleus. Notably, cyanine dye **5** appears to stain a specific organelle within the nucleoplasm but does not localize in the DNA. In contrast, cyanine dye **10** displays a significantly lower fluorescence intensity compared to dye **5**. Additionally, the fluorescence emitted by dye **10** diminishes over time, with a noticeable loss of intensity.

Staining with Hoescht 33342 enabled the observation of nuclear morphology, and the results from this assay are consistent with the findings from optical microscopy. Both cyanine dyes exhibited nuclei similar to those of untreated cells. However, identifying cells in mitosis (as indicated by DNA staining) was challenging, as noted in the previous assay. Additionally, both dyes resulted in fewer cells per slide compared to the control, with cyanine **5** showing the lowest cell density. Therefore, it can be qualitatively inferred that both dyes reduce the cell count per slide, potentially inhibiting cell proliferation. However, an accurate determination of their mechanisms of action remains necessary, and a certain degree of cell death cannot be ruled out, as dead cells may have been lost during the washing stages.

#### 2.2.4. Apoptosis Induction and Cell Cycle Arrest Studies by Flow Cytometry

**Figure 6:**
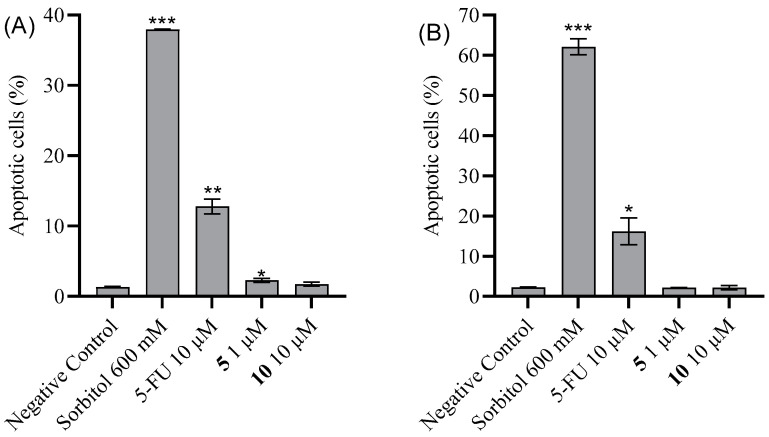
Analysis of apoptosis in Caco-2 cells treated with positive controls 5-FU (10 µM) and sorbitol (600 mM) and with cyanine dyes **5** (1 µM) and **10** (10 µM), after 72 (**A**) and 120 h (**B**) of incubation. Sorbitol was incubated for 24 h. Results are expressed as average values ± standard deviation of two independent assays performed in duplicate. (*) *p* < 0.05, (**) *p* < 0.01 or (***) *p* < 0.001 versus negative control in the statistical significance analysis (Student’s *t*-test).

Given that apoptosis is a common programmed mechanism regulating cell death, characterized by DNA fragmentation and an increase in sub-G1 events, we employed the method described by Riccardi and Nicolleti [20] to assess potential apoptosis induction using flow cytometry. Cyanine dyes **5** and **10** were tested at concentrations of 1 µM and 10 µM, respectively. As positive controls for apoptosis, 600 mM sorbitol and 10 µM 5-FU were used, with untreated cells serving as the negative control. The results showed that dye **5** induced apoptosis in approximately 2.3% of cells after 72 h of treatment (Figure 6A), representing a modest but significant increase from the 1.3% observed in untreated cells. In contrast, dye **10** induced a slight increase in apoptosis, reaching 1.7%. Although not as pronounced as the effects of the positive control agents, sorbitol and 5-FU, which induced apoptosis in 37.9% and 12.8% of cells, respectively, the apoptosis induction observed for the cyanine dyes aligns with the findings from optical microscopy. Apoptotic bodies were more evident in cells treated with dye **5** compared to dye **10**, which is further supported by the slightly higher apoptosis induction in this assay. Additionally, extending the treatment with these dyes to 48 h (Figure 6B) did not yield significant changes in the results.

After excluding sub-G1 events, we proceeded to evaluate the impact of dyes 5 and 10 on cell cycle distribution. Since previous studies have shown that sorbitol does not significantly affect the Caco-2 cell cycle [17], only 5-FU at a concentration of 10 μM was used as a positive control.

**Figure 7:**
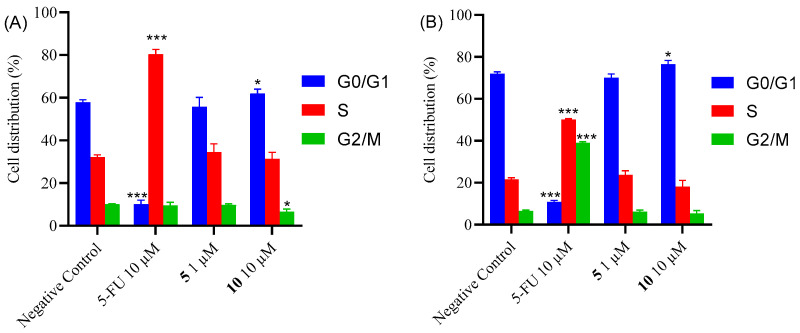
Analysis of cell cycle distribution of Caco-2 cells treated with positive controls 5-FU (10 µM) and with monomethine cyanine dyes **5** (1 µM) and **10** (10 µM) after 72 (**A**) and 120 h (**B**) of incubation. Results are expressed as average values ± standard deviation of two independent assays performed in duplicate. (*) *p* < 0.05 or (***) *p* < 0.001 versus negative control in the statistical significance analysis (Student’s *t*-test).

As shown in Figure 7, treatment with dye **5** did not significantly alter the cell cycle distribution of Caco-2 cells, though this does not rule out an effect on cell proliferation. In fact, cells may progress more slowly through the cell cycle without arresting at a specific phase. Additionally, Caco-2 cell proliferation is inhibited by increased cell density, as evidenced by a reduction in S phase cells in untreated samples from 74 to 120 h. Interestingly, a similar decrease in proliferation was observed in dye **5**-treated cells, despite their much lower density.

In contrast, cells treated with cyanine dye **10** showed a significant increase in the proportion of cells in the G0/G1 phase, reaching approximately 62.0% at 72 h (Figure 7A), accompanied by a decrease in G2/M phase cells from 10.1% to 6.6%. These results correspond with the earlier-discussed morphological changes. The cellular enlargement induced by 5-FU, which suggests stalling in the S phase, is confirmed here by an increase in the proportion of cells in this phase to around 80.3%, with a concurrent reduction in G0/G1 phase cells to 10.1%, as expected [17]. On the other hand, the contraction and reduction in cell size induced by dye **10** can be partially explained by the observed mild arrest in the G0/G1 phase.

#### 2.2.5. Topoisomerase II Inhibition

**Figure 8:**
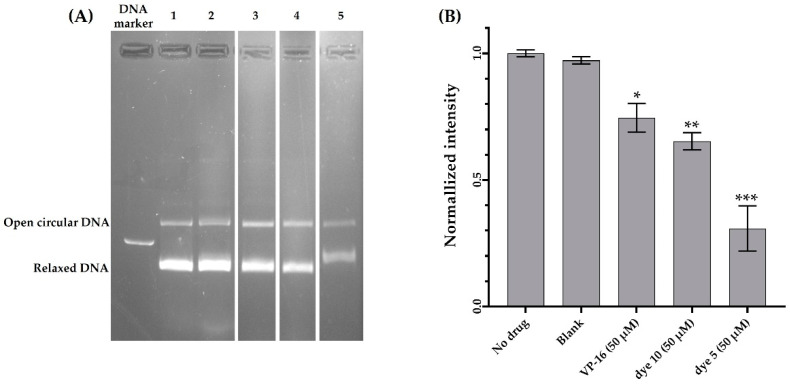
Results. (**A**) Agarose gel electrophoresis results displaying the effect of cyanines on topoisomerase II. Lane DNA marker: Linear pRYG DNA marker; Lane 1: includes pRYG DNA and the enzyme Topo II (“No drug” sample/Negative control); Lane 2: includes kDNA, Topo II and solvent (DMSO:Tris-HCl—1:9) used in sample preparation (“Blank” sample); Lane 3: includes pRYG DNA and Topo II in the presence of 50 µM VP-16 (etoposide), which is a proven Topo II poison (Positive control); Lane 4: pRYG DNA and Topo II in the presence of 50.0 µM cyanine dye **10**; Lane 5: pRYG DNA and Topo II in the presence of 50.0 µM cyanine dye **5**. In all samples, 5.0 units of enzyme and/or 250.0 ng of pRYG DNA were used. (**B**) Normalized band intensities for the selected relaxed DNA band were obtained by analyzing the image in (**A**). Normalization was performed based on the relaxed DNA band intensity in the negative control sample (“No drug” sample—Lane 1), which only includes the substrate (pRYG DNA) and the enzyme (Topo II). Band intensities were measured using the software Image Lab, Version 6.1 (BioRad Laboratories). Results are expressed as average values ± standard deviation of two independent assays. (*) *p* < 0.05; (**) *p* < 0.01 or (***) *p* < 0.001 versus “No drug” sample (relaxed DNA band of Lane 1).

Since several azacyanines have been previously identified as topoisomerase II (Topo II) inhibitors [21], and considering that Topo II is a relevant target for many anticancer drugs in clinical use (e.g., doxorubicin) [22], we evaluated the effects of the most promising cyanines on this target (Figure 8). To do so, the impact of cyanines on Topo II activity was assessed using a pRYG DNA substrate in the presence and absence of the dyes. This was monitored via a Topo II drug screening kit followed by agarose gel electrophoresis. The agarose gel image in Figure 8A illustrates the effect of 50 µM VP-16 and cyanine dyes **5** and **10** on Topo II. All samples applied to the agarose gel contained 5.0 units of Topo II and 250.0 ng of pRYG DNA as the substrate. Linear pRYG DNA (Lane DNA marker) served as the reference control for the DNA band positions, and 50 µM VP-16 (Lane 3) was used as a positive control. A “blank sample” (Lane 2) containing a DMSO (1:9) mixture, which was used as a solvent for the preparation of VP-16, dye **5**, and dye **10** stock solutions, was included to assess any potential effect of the solvent on Topo II activity.

The formation of relaxed DNA products, indicating Topo II activity, is visible in Lane 1 (incubation of pRYG DNA with Topo II) of Figure 8A. As expected, the intensity of the relaxed DNA bands decreased in the presence of VP-16 (positive control—Lane 3), due to Topo II inhibition. A similar reduction in the intensity of the relaxed DNA bands was observed in the presence of dyes **5** and **10** (Lanes 4 and 5 of Figure 8A). The intensity of the relaxed DNA bands from two independent experiments was quantified for all samples and normalized to the Topo II activity in the absence of inhibitors (No drug sample, Lane 1) using Image Lab, Version 6.1 (Figure 8B). The normalization was based on the “No drug” sample (Lane 1), which reflects full Topo II activity without inhibition. The relative intensity of the “Blank” sample was comparable to the “No drug” sample, confirming that the solvent used in stock solution preparation had no significant effect on Topo II activity.

In contrast, the intensity of the relaxed DNA band decreased to 0.75 ± 0.04 in the presence of 50 µM VP-16, indicating Topo II inhibition. Interestingly, the inhibitory effect of 50 µM cyanine dyes **5** and **10** on Topo II was significantly greater than that of VP-16, with relative intensities of 0.65 ± 0.024 (*p* < 0.01) for dye **10** and 0.31 ± 0.063 (*p* < 0.001) for dye **5** (Figure 8B). These findings demonstrate that both cyanines exhibit higher Topo II inhibitory activity than the chemotherapeutic agent VP-16, particularly dye **5**. Notably, a previous study on azacyanines reported the highest activity for a derivative also methylated at the cyanine nitrogen [21].

Furthermore, the formation of a linear DNA product (band presented in the DNA marker lane), typically seen in the presence of strong Topo II poisons, was not observed with the cyanine dyes. Similar to azacyanines, the reduction in relaxed DNA product intensity without the formation of a linear product suggests that these cyanines function as catalytic inhibitors rather than poisons [22].

### 2.3. In Silico Drug-Likeness Predictions

One of the primary challenges in drug candidate development is the limited efficacy or poor pharmacokinetic profiles [23]. To mitigate these issues, the online tool SwissADME [24] was used to analyze the molecular properties of the compounds with the highest potential in this study, specifically, cyanine dyes **5** and **10**. The goal was to predict their prospective in vivo applicability. Table 3 presents key molecular characteristics, including the number of hydrogen bond donors or acceptors, rotatable bonds, molecular weight (MW), the logarithm of the 1-octanol/water partition coefficient (CLogP), molar refractivity, and topological polar surface area (TPSA), as the latter being an important parameter for predicting intestinal absorption and oral bioavailability.

These parameters are well-established predictors, initially described through Lipinski’s “rule of five” [25], and later refined by other guidelines, including those by Ghose [26], Veber [27], Egan [28], and Muegge [29]. Given that cyanine dyes **5** and **10** meet all these criteria, they comply with the drug-likeness filters. As a result, both compounds show promise as drug-like substances, with favorable properties for intestinal absorption and oral bioavailability.

To anticipate potential issues such as biological promiscuity, high reactivity, and instability, factors that could suggest a nonspecific mode of action for cyanine dyes **5** and **10**, medicinal chemistry filters using the SwissADME tool to identify potentially problematic fragments were used. The results in Table 3 indicate that dyes **5** and **10** are unlikely to be pan-assay interference compounds (PAINS) [30]. However, the Brenk filter [31] triggered an alert due to the presence of quaternary nitrogen fragments. While this fragment is sometimes associated with toxicity issues [32], it is important to note that the positive charge is only partially present, as it is delocalized between the two nitrogen atoms and across the polymethine chain.

Additionally, dye **10** triggered an additional warning due to the presence of a nitro group, which is often associated with increased toxicity [33]. Moreover, dye **10** does not meet the lead-likeness criteria, indicating that it may not be suitable as a lead compound. Taking all these alerts into consideration, along with the in vitro findings discussed earlier, we emphasize that cyanine dye **5** stands out as a more promising candidate for further investigation compared to dye **10**.

## 3. Materials and Methods

### 3.1. Chemistry

All reagents and solvents were purchased from commercial suppliers and used without further purification. 2-Methyl-6-nitrobenzothiazole and 5-acetamido-2-methylbenzothiazole were prepared as previously described [34]. 1-Alkyl-2-methylthiobenzoazolium, 1-alkyl-2-methylthioquinolinium, 1-alkyl-2-methylbenzoazolium and 1-alkyl-2-methylquinolinium salts and their respective heteroaryl-substituted derivatives were prepared according to the previously described method [34,35]. All reactions were monitored by TLC using 60 G/UV_254_ (0.2 mm) plates (Macherey-Nagel, Durën, Germany), which were eluted in dichloromethane or dichloromethane/methanol (9:1), and were visualized under UV radiation at 254 nm. m.p. were measured on a B-540 apparatus (Büchi, Flawil, Switzerland) and are uncorrected. UV-Vis spectra were recorded for all dyes under study on an Evolution 160 UV-VIS spectrophotometer (Thermo Scientific, Madison, WI, USA) using ethanol as a solvent. The wavelength of maximum absorption is reported in nm. NMR spectra were acquired on an Brüker (Fitchburg, WI, USA) Avance III 400 MHz spectrometer (^1^H NMR at 400.13 MHz and ^13^C NMR at 100.62 MHz) and were processed with the software MestReNova 14.2.0 lite. The chemical shift (δ) values are given in parts per million (ppm) and coupling constants (*J*) in Hertz (Hz). The multiplicity of the signals is reported as singlet (s), doublet (d), doublet of doublets (dd), doublet of triplets (dt), doublet of doublet of doublets (ddd), triplet (t), triplet of doublets (td), quartet (q), pentet (p), or multiplet (m). Dimethyl sulfoxide hexadeutered (DMSO-*d*_6_) was used as a solvent and internal standard (2.50 and 39.52 ppm in ^1^H and ^13^C NMR, respectively). HRMS was performed for new compounds by electrospray ionization time-of-flight (ESI-TOF) at NUCLEUS services at the University of Salamanca (Spain).

General procedures (GP) for the synthesis of monomethine cyanine dyes **1**–**12**.

Following our previous study [17], cyanine dyes **1**–**7**, **9**, **10**, **12** were prepared accordingly following the thioalkyl method by one-pot synthesis (GP1), the thioalkyl method (GP3), or the potassium hydroxide method (GP4). Furthermore, and as described below, cyanine **8** was obtained from acetamidocyanine **7** by acid hydrolysis and cyanine **11** from nitrocyanine **10** by reduction.

*3-Methyl-2-((3-pentylbenzo[d]thiazol-2(3H)-ylidene)methyl)benzo[d]thiazol-3-ium iodide* (**1**), GP2 method, from 3-methyl-2-(methylthio)benzo[*d*]thiazol-3-ium iodide (323 mg) and 2-methyl-3-pentylbenzo[*d*]thiazol-3-ium iodide (347 mg) in ethanol. Yield: 72%; yellow crystals; m.p. 254–258 °C; Vis λ_max_ (EtOH): 425 nm, log ε = 4.85; ^1^H NMR (400 MHz, DMSO-*d*_6_): δ 8.21 (d, *J* = 8.0 Hz, 2H), 7.88 (d, *J* = 8.4 Hz, 2H), 7.68 (t, *J* = 7.8 Hz, 2H), 7.50 (t, *J* = 7.6 Hz, 2H), 6.70 (s, 1H), 4.62 (t, *J* = 7.5 Hz, 2H), 4.03 (s, 3H), 1.81 (p, *J* = 7.7 Hz, 2H), 1.54–1.28 (m, 4H), 0.88 (t, *J* = 7.0 Hz, 3H) ppm; ^13^C NMR (101 MHz, DMSO-*d*_6_): δ 162.0, 161.4, 140.6, 140.1, 128.5, 128.4, 124.9, 124.8, 124.8, 124.7, 123.5, 123.4, 113.8, 82.6, 46.2, 34.3, 28.1, 26.5, 21.9, 13.8 ppm; ESI-HRMS: Calculated for [M − I]^+^ C_21_H_23_N_2_S_2_^+^ 367.1297, found 367.1289.
*3-Methyl-2-((3-decylbenzo[d]thiazol-2(3H)-ylidene)methyl)benzo[d]thiazol-3-ium iodide* (**2**), GP2 method, from 3-methyl-2-(methylthio)benzo[*d*]thiazol-3-ium iodide (323 mg) and 3-decyl-2-methylbenzo[*d*]thiazol-3-ium iodide (417 mg) in ethanol. Yield: 68%; yellow crystals; m.p. 217–218 °C; Vis λ_max_ (EtOH): 427 nm, log ε = 4.95; ^1^H NMR (400 MHz, DMSO-*d*_6_): δ 8.22 (d, *J* = 8.1 Hz, 2H), 7.89 (d, *J* = 8.3 Hz, 2H), 7.69 (t, *J* = 7.7 Hz, 1H), 7.68 (t, *J* = 7.7 Hz, 1H), 7.50 (t, *J* = 7.6 Hz, 2H), 6.70 (s, 1H), 4.63 (t, *J* = 7.4 Hz, 2H), 4.02 (s, 3H), 1.80 (p, *J* = 7.8 Hz, 2H), 1.43 (p, *J* = 7.0 Hz, 2H), 1.32 (p, *J* = 6.3 Hz, 2H), 1.36–1.15 (m, 10H), 0.82 (t, *J* = 6.8 Hz, 3H) ppm; ^13^C NMR (101 MHz, DMSO-*d*_6_): δ 162.1, 161.4, 140.6, 140.1, 128.5, 128.4, 124.9, 124.8, 124.8, 124.7, 123.5, 123.4, 113.8, 113.8, 82.7, 46.2, 34.2, 31.2, 28.9, 28.8, 28.7, 28.6, 26.8, 25.9, 22.0, 13.9 ppm; ESI-HRMS: Calculated for [M − I]^+^ C_26_H_33_N_2_S_2_^+^ 437.2080, found 437.2071.
*3-Methyl-2-((3-methylbenzo[d]oxazol-2(3H)-ylidene)methyl)benzo[d]thiazol-3-ium 4-methylbenzenesulfonate* (**3**), GP1 method, from 2-(methylthio)benzothiazole (181 mg), 2-methylbenzoxazole (133 mg) and methyl *p*-toluenesulfonate (409 mg). Yield: 47%; yellow crystals; m.p. 270–273 °C (lit. 301–304 °C [19]); Vis λ_max_ (EtOH): 401 nm, log ε = 4.88; ^1^H NMR (400 MHz, DMSO-*d*_6_): δ 8.11 (d, *J* = 7.9 Hz, 1H), 7.81 (d, *J* = 9.2 Hz, 2H), 7.71 (d, *J* = 7.8 Hz, 1H), 7.64 (t, *J* = 7.8 Hz, 1H), 7.54 (t, *J* = 7.7 Hz, 1H), 7.50–7.42 (m, 4H), 7.10 (d, *J* = 7.8 Hz, 2H), 6.28 (s, 1H), 3.98 (s, 3H), 3.84 (s, 3H), 2.28 (s, 3H) ppm; ^13^C NMR (101 MHz, DMSO-*d*_6_): δ 163.4, 161.3, 146.1, 145.7, 140.5, 137.7, 131.4, 128.1, 128.0, 126.3, 125.5, 125.1, 124.9, 124.8, 123.0, 113.5, 111.4, 110.8, 69.8, 34.0, 30.6, 20.8 ppm.
*1,3-Dimethyl-2-((3-methylbenzo[d]thiazol-2(3H)-ylidene)methyl)-1H-benzo[d]imidazol-3-ium iodide* (**4**), GP1 method, from 2-(methylthio)benzothiazole (352 mg), 2-methyl-1*H*-benzo[*d*]imidazole (132 mg) and methyl *p*-toluenesulfonate (596 mg). Anion exchange was made by reflux in a saturated potassium iodide solution (10 mL). Yield: 84%; brown powder; m.p. 270–272 °C (287–289 °C in the form of perchlorate anion [36]); Vis λ_max_ (EtOH): 385 nm, log ε = 5.26; ^1^H NMR (400 MHz, DMSO-*d*_6_): δ 7.91–7.82 (m, 2H), 7.75 (dd, *J* = 7.6, 1.0 Hz, 1H), 7.63–7.54 (m, 2H), 7.52–7.41 (m, 2H), 7.22 (ddd, *J* = 8.3, 6.9, 1.6 Hz, 1H), 5.79 (s, 1H), 3.90 (s, 6H), 3.70 (s, 3H) ppm; ^13^C NMR (101 MHz, DMSO-*d*_6_): δ 158.6, 151.4, 141.4, 132.4, 127.3, 125.3, 123.0, 122.6, 122.3, 112.1, 111.3, 68.5, 33.3, 32.8 ppm.
*3-Methyl-2-((1-methylquinolin-2(1H)-ylidene)methyl)benzo[d]oxazol-3-ium 4-methylbenzenesulfonate* (**5**), GP1 method, from 2-(methylthio)benzoxazole (165 mg), quinaldine (143 mg) and methyl *p*-toluenesulfonate (409 mg). Yield: 63%; Orange crystals; m.p. 243–246 °C; Vis λ_max_ (EtOH): 436 nm, log ε = 4.72; ^1^H NMR (400 MHz, DMSO-*d*_6_): δ 8.39–8.31 (m, 2H), 8.10 (d, *J* = 8.8 Hz, 1H), 7.99 (dd, *J* = 7.8, 1.6 Hz, 1H), 7.87 (ddd, *J* = 8.8, 7.1, 1.6 Hz, 1H), 7.81 (d, *J* = 8.0 Hz, 1H), 7.67 (dd, *J* = 8.0, 1.2 Hz, 1H), 7.58 (t, *J* = 7.4 Hz, 1H), 7.54–7.44 (m, 1H), 7.47 (d, *J* = 8.1 Hz, 2H), 7.41 (td, *J* = 7.8, 1.3 Hz, 1H), 7.10 (d, *J* = 7.8 Hz, 2H), 5.62 (s, 1H), 4.10 (s, 3H), 3.83 (s, 3H), 2.28 (s, 3H) ppm; ^13^C NMR (101 MHz, DMSO-*d*_6_): δ 161.3, 153.3, 145.8, 145.7, 139.3, 138.6, 137.6, 132.8, 131.2, 129.2, 128.0, 125.9, 125.5, 125.3, 124.5, 123.7, 119.8, 117.1, 111.0, 110.8, 72.4, 37.5, 30.6, 20.8 ppm.
*3-Methyl-2-((1-methylquinolin-2(1H)-ylidene)methyl)benzo[d]thiazol-3-ium 4-methylbenzenesulfonate* (**6**), by GP1 method, from 2-(methylthio)benzothiazole (181 mg), quinaldine (143 mg) and methyl *p*-toluenesulfonate (409 mg). Yield: 72%; orange reddish crystals; m.p. 255–257 °C (lit. 272–276 °C in the form of iodide anion [37]); Vis λ_max_ (EtOH): 483 nm, log ε = 4.53; ^1^H NMR (400 MHz, DMSO-*d*_6_): δ 8.44 (d, *J* = 9.4 Hz, 1H), 8.13 (d, *J* = 8.8 Hz, 1H), 8.07 (d, *J* = 9.2 Hz, 1H), 8.02 (d, *J* = 7.9 Hz, 2H), 7.90 (t, *J* = 8.1 Hz, 1H), 7.78 (d, *J* = 8.4 Hz, 1H), 7.61 (t, *J* = 8.0 Hz, 2H), 7.47 (d, *J* = 7.7 Hz, 2H), 7.42 (t, *J* = 7.6 Hz, 1H), 7.10 (d, *J* = 7.7 Hz, 2H), 6.16 (s, 1H), 4.12 (s, 3H), 3.94 (s, 3H), 2.28 (s, 3H) ppm; ^13^C NMR (101 MHz, DMSO-*d*_6_): δ 161.4, 153.1, 145.7, 140.6, 139.7, 139.6, 137.6, 133.3, 129.4, 128.1, 128.0, 125.6, 125.5, 124.8, 123.9, 123.4, 122.8, 118.4, 117.3, 113.0, 86.5, 37.6, 33.9, 20.8 ppm.
*5-Acetamido-3-ethyl-2-((3-ethylbenzo[d]thiazol-2(3H)-ylidene)methyl)benzo[d]thiazol-3-ium 4-methylbenzenesulfonate* (**7**), GP1 method, from 2-(ethylthio)benzothiazole (195 mg), 5-acetamido-2-methylbenzothiazole (206 mg) and ethyl *p*-toluenesulfonate (440 mg). Yield: 29%; yellow crystals; m.p. 278–279 °C; Vis λ_max_ (EtOH): 432 nm, log ε = 4.92; ^1^H NMR (400 MHz, DMSO-*d*_6_): δ 10.40 (s, 1H), 8.25 (d, *J* = 1.8 Hz, 1H), 8.21 (dd, *J* = 8.1, 1.2 Hz, 1H), 8.10 (d, *J* = 8.7 Hz, 1H), 7.89 (d, *J* = 8.4 Hz, 1H), 7.68 (ddd, *J* = 8.5, 7.3, 1.2 Hz, 1H), 7.54–7.45 (m, 2H), 7.47 (d, *J* = 8.1 Hz, 2H), 7.10 (d, *J* = 7.7 Hz, 2H), 6.74 (s, 1H), 4.69 (q, *J* = 7.2 Hz, 2H), 4.58 (q, *J* = 7.2 Hz, 2H), 2.28 (s, 3H), 2.11 (s, 3H), 1.39 (t, *J* = 7.4 Hz, 3H), 1.37 (t, *J* = 7.5 Hz, 3H) ppm; ^13^C NMR (101 MHz, DMSO-*d*_6_): δ 169.1, 162.2, 161.2, 145.8, 140.2, 140.2, 139.8, 137.5, 128.7, 128.0, 125.5, 125.1, 125.0, 123.8, 123.6, 118.7, 116.4, 113.6, 103.2, 82.1, 41.7, 41.6, 24.2, 20.8, 12.3, 12.1 ppm; ESI-HRMS: Calculated for [M − TsO]^+^ C_21_H_22_N_3_OS_2_^+^ 396.1199, found 396.1194.
*5-Amino-3-ethyl-2-((3-ethylbenzo[d]thiazol-2(3H)-ylidene)methyl)benzo[d]thiazol-3-ium iodide* (**8**), 5-acetamidocyanine dye **7** (1.0 mmol, 568 mg) was refluxed in a 25% hydrochloric acid solution (5 mL) for a duration of 30 min. The mixture was cooled to room temperature and neutralized with a 25% ammonia solution. The obtained solid was filtered under reduced pressure and anion exchange was made by reflux in a saturated potassium iodide solution (10 mL). Yield: 31%; green crystals; m.p. 288–289 °C; Vis λ_max_ (EtOH): 454 nm, log ε = 4.67; ^1^H NMR (400 MHz, DMSO-*d*_6_): δ 8.18 (d, *J* = 7.9 Hz, 1H), 7.84 (d, *J* = 8.4 Hz, 1H), 7.79 (d, *J* = 8.6 Hz, 1H), 7.66 (t, *J* = 7.8 Hz, 1H), 7.46 (t, *J* = 7.7 Hz, 1H), 6.89 (s, 1H), 6.77 (d, *J* = 8.4 Hz, 1H), 6.69 (s, 1H), 5.80 (s, 2H), 4.66 (q, *J* = 7.1 Hz, 2H), 4.53 (q, *J* = 7.4 Hz, 2H), 1.37 (t, *J* = 7.2 Hz, 3H), 1.36 (t, *J* = 7.3 Hz, 3H) ppm; ^13^C NMR (101 MHz, DMSO-*d*_6_): δ 161.8, 160.0, 150.3, 141.2, 139.8, 128.5, 125.0, 124.6, 123.6, 123.5, 113.3, 113.0, 110.7, 96.6, 81.6, 41.5, 41.3, 12.2 ppm; ESI-HRMS: Calculated for [M − I]^+^ C_19_H_20_N_3_S_2_^+^ 354.1093, found 354.1086.
*1,3-Diethyl-2-((3-ethylbenzo[d]thiazol-2(3H)-ylidene)methyl)-5-nitro-1H-benzo[d]imidazol-3-ium iodide* (**9**), GP1 method, from 2-(ethylthio)benzothiazole (195 mg), 2-methyl-5-nitrobenzimidazole (177 mg) and ethyl *p*-toluenesulfonate (700 mg). Anion exchange was made by reflux in a saturated potassium iodide solution (10 mL). Yield: 26%; Red crystals; m.p. 219–220 °C; Vis λ_max_ (EtOH): 422 nm, log ε = 4.56; ^1^H NMR (400 MHz, DMSO-*d*_6_): δ 8.92 (d, *J* = 2.1 Hz, 1H), 8.44 (dd, *J* = 9.0, 2.2 Hz, 1H), 8.17 (d, *J* = 9.0 Hz, 1H), 7.81 (dd, *J* = 7.8, 0.9 Hz, 1H), 7.54 (d, *J* = 8.1 Hz, 1H), 7.48 (td, *J* = 8.2, 7.4, 0.9 Hz, 1H), 7.26 (td, *J* = 7.6, 0.8 Hz, 1H), 5.88 (s, 1H), 4.59 (q, *J* = 7.1 Hz, 2H), 4.52 (q, *J* = 7.2 Hz, 2H), 4.35 (q, *J* = 7.0 Hz, 2H), 1.39–1.30 (m, 9H) ppm; ^13^C NMR (101 MHz, DMSO-*d*_6_): δ 158.5, 153.8, 144.7, 140.4, 135.9, 131.6, 127.6, 123.4, 122.9, 122.6, 120.8, 113.3, 111.6, 109.1, 67.6, 42.2, 42.1, 40.4, 14.0, 13.8, 11.5 ppm; ESI-HRMS: Calculated for [M − I]^+^ C_21_H_23_N_4_O_2_S^+^ 395.1536, found 395.1527.
*3-Ethyl-2-((3-ethylbenzo[d]thiazol-2(3H)-ylidene)methyl)-6-nitrobenzo[d]thiazol-3-ium 4-methylbenzenesulfonate* (**10**), by GP1 method, from 2-(ethylthio)benzothiazole (195 mg), 2-methyl-6-nitrobenzothiazole (177 mg) and ethyl *p*-toluenesulfonate (700 mg). Yield: 53%; orange crystals; m.p. 313–314 °C; Vis λ_max_ (EtOH): 439 nm, log ε = 4.81; ^1^H NMR (400 MHz, DMSO-*d*_6_): δ 9.21 (d, *J* = 2.4 Hz, 1H), 8.49 (dd, *J* = 9.1, 2.4 Hz, 1H), 8.33 (dd, *J* = 8.1, 1.2 Hz, 1H), 8.05–7.95 (m, 2H), 7.75 (ddd, *J* = 8.4, 7.2, 1.3 Hz, 1H), 7.58 (t, *J* = 7.6 Hz, 1H), 7.47 (d, *J* = 8.1 Hz, 2H), 7.10 (d, *J* = 7.8 Hz, 2H), 6.85 (s, 1H), 4.77 (q, *J* = 7.5 Hz, 2H), 4.71 (q, *J* = 7.5 Hz, 2H), 2.28 (s, 3H), 1.39 (t, *J* = 7.1 Hz, 3H), 1.38 (t, *J* = 7.1 Hz, 3H) ppm; ^13^C NMR (101 MHz, DMSO-*d*_6_): δ 162.8, 162.7, 145.8, 144.6, 143.4, 139.8, 137.6, 129.0, 128.1, 126.1, 125.7, 125.6, 125.5, 124.4, 124.0, 120.0, 114.3, 113.5, 83.9, 42.2, 42.0, 20.8, 12.6, 12.2 ppm; ESI-HRMS: Calculated for [M–TsO]^+^ C_19_H_18_N_3_O_2_S_2_^+^ 384.0835, found 384.0828.
*6-Amino-3-ethyl-2-((3-ethylbenzo[d]thiazol-2(3H)-ylidene)methyl)benzo[d]thiazol-3-ium iodide* (**11**), hydrazine hydrate (3 mmol, 148 µL) was added to a solution of 6-nitrocyanine **10** (1 mmol, 556 mg) in a minimal amount of etilenoglicol,. After heating at 80 °C for 5 min, a few drops of a 50% Raney nickel solution were added. The resulting mixture was refluxed for an additional 6 h, hot filtered, and a saturated potassium iodide solution was added to the filtrate. After cooling, the obtained solid was filtered, dried, and recrystallized from methanol. Yield: 81%; Brown crystals; m.p. 233–235 °C (described without m.p. [18]); Vis λ_max_ (EtOH): 444 nm, ε = 4.62; ^1^H NMR (400 MHz, DMSO-*d*_6_): δ 8.10 (d, *J* = 7.8 Hz, 1H), 7.75 (d, *J* = 8.2 Hz, 1H), 7.67–7.49 (m, 2H), 7.39 (t, *J* = 7.6 Hz, 1H), 7.22 (d, *J* = 2.4 Hz, 1H), 6.88 (dd, *J* = 8.7, 2.3 Hz, 1H), 6.54 (s, 1H), 5.67 (s, 2H), 4.57 (q, *J* = 7.4 Hz, 4H), 1.33 (t, *J* = 7.0 Hz, 6H) ppm; ^13^C NMR (101 MHz, DMSO-*d*_6_): δ 159.2, 158.5, 147.4, 139.9, 130.1, 128.4, 126.8, 124.5, 124.3, 123.3, 115.6, 114.4, 112.9, 105.5, 81.1, 41.7, 41.1, 12.5, 12.0 ppm.
*1-Methyl-4-((1-methylquinolin-2(1H)-ylidene)methyl)quinolin-1-ium 4-methylbenzenesulfonate* (**12**), GP3 method, from 1,2-dimethylquinolin-1-ium 4-methylbenzenesulfonate (329 mg), and 1-methylquinolin-1-ium 4-methylbenzenesulfonate (315 mg). Yield: 68%; dark blue crystals; m.p. 199–201 °C (lit. 268 °C in the form of iodide anion [38]); Vis λ_max_ (EtOH): 558 nm, log ε = 5.26; ^1^H NMR (400 MHz, DMSO-*d*_6_): δ 8.56 (d, *J* = 8.5 Hz, 1H), 8.23 (d, *J* = 7.2 Hz, 1H), 8.08 (d, *J* = 9.4 Hz, 1H), 8.02–7.92 (m, 4H), 7.89 (dd, *J* = 7.9, 1.6 Hz, 1H), 7.79 (ddd, *J* = 8.8, 7.1, 1.6 Hz, 1H), 7.67 (dt, *J* = 8.3, 4.1 Hz, 1H), 7.50 (t, *J* = 7.2 Hz, 1H), 7.47 (d, *J* = 8.1 Hz, 2H), 7.40 (d, *J* = 7.2 Hz, 1H), 7.10 (d, *J* = 7.8 Hz, 2H), 6.49 (s, 1H), 4.07 (s, 3H), 4.01 (s, 3H), 2.28 (s, 3H) ppm; ^13^C NMR (101 MHz, DMSO-*d*_6_): δ 153.9, 149.3, 145.8, 143.5, 140.0, 138.4, 137.6, 137.1, 132.8, 132.4, 128.8, 128.0, 126.3, 125.8, 125.5, 124.8, 124.4, 124.3, 121.2, 117.6, 116.6, 108.5, 94.4, 41.7, 37.7, 20.8 ppm.

### 3.2. In Vitro Studies

A dimethyl sulfoxide stock solution of each compound under study at 1 mM or 10 mM was prepared and used. These solutions were stored at 4 °C and in the absence of light.

#### 3.2.1. Cytotoxic Effects in Human Cell Lines

##### Cell Culture

The cell lines used in this study (Caco-2, MCF-7, PC-3, and NHDF) were obtained from the American Type Culture Collection (ATCC, Manassas, VA, USA) and cultured in 75 cm^2^ culture flasks at 37 °C in a humidified atmosphere with 5% CO_2_ in a LEEC Culture Safe Precision CO_2_ Incubator. PC-3 cells were grown in Roswell Park Memorial Institute (RPMI) medium supplemented with 10% of inactivated Fetal Bovine Serum (FBS) and 1% of an antibiotic mixture (Sp; 10,000 U/mL of penicillin G and 100 mg/mL of streptomycin). MCF-7 and Caco-2 cell lines were both cultured using Dulbecco’s Modified Eagle Medium (DMEM). However, the medium for MCF-7 cells was complemented with 10% of inactivated FBS and 1% of antibiotic/antimycotic mixture (Ab; 10,000 U/mL of penicillin G, 100 mg/mL of streptomycin and 25 µg/mL of amphotericin B), and the medium for Caco-2 cells was supplemented with 20% of inactivated FBS and 1% of Sp. Finally, RPMI medium supplemented with 10% FBS, 2 mM of *L*-glutamine, 10 mM of 4-(2-hydroxyethyl)-1-piperazineethanesulfonic acid (HEPES), 1 mM of sodium pyruvate, and 1% of Ab was used to culture NHDF cells. The culture media were renewed every two to three days until near confluence was achieved. Then, cells were detached using a solution of 0.125 g/L of trypsin and 0.02 g/L of ethylenediaminetetracetic acid (EDTA), counted by the trypan blue method, resuspended, and adequately seeded to continue the culture in 75 cm^2^ flasks or for the experiments described below. All manipulations were performed in a Labculture Class II A2 Biological Safety Cabinet.

##### MTT Assay

After trypsinization, cells were seeded in 96-well culture plates (100 µL; 2 × 10^4^ cells/mL), and after 48 h (for adherence), they were incubated with the compounds under study in the absence of light for a duration of 72 h. Then, the medium was removed, and fresh incomplete culture medium (without FBS and Ab or Sp) and MTT solution [5 mg/mL in phosphate buffer saline (PBS)] were added to each well, followed by 4 h of incubation. After this, the medium containing MTT was removed and DMSO was added to dissolve formazan crystals. The absorbance of each well at 570 nm was determined using a microplate spectrophotometer Bio-Rad (Hercules, CA, USA) xMark. Untreated cells were considered the negative control, and 5-FU, a clinically used drug, was considered the positive control. For data analysis, the relative cell proliferation relative to the negative control cells was calculated. For each cell viability study, at least two independent assays in quadruplicate were performed. Increasing concentrations of the compounds between 0.001 and 100 μM were used to determine the IC_50_ values.

##### Phototoxicity Assay

A phototoxicity evaluation was performed on the NHDF cell line with a similar methodology to the MTT assay and following previously validated methods [17]. After 48 h of incubation with increasing concentrations ranging from 0.001 to 100 μM, cells were subjected to 30 min of constant irradiation with the white light of a Luxtar (Yancheng, Jiangsu, China) RGBW LED projector (220–240 V and 30 W). After this irradiation, the cells were incubated at 37 °C for an additional 24 h (72 h in total). This assay was performed in quadruplicate, and two independent assays were realized.

##### Photostability Assay

In a 96-well plate, 100 µL of dyes **5** and **10** at a concentration of 10 µM, prepared from 10 mM DMSO stock solution diluted in PBS, was added at each well. The photostability assay was performed by irradiating the as previously described for photocytotoxicity evaluation, repeated several times up to 120 min. Spectrophotometric readings were taken over the maximum absorption wavelength of each dye (436 nm for **5** and 439 nm for **10**). Two independent experiments were performed in quadruplicate, and the obtained absorbance values were normalized.

##### Morphological Analysis

Caco-2 cells near confluency were trypsinized, counted, and then seeded at 3 × 10^4^ cells/mL (3 mL) in 35 mm cell culture dishes previously treated for tissue culture. After cell adherence (48 h), they were incubated with solutions under study for 72 h in the absence of light. The cellular morphology was verified every 24 h using a CKX41 SF inverted microscope (Olympus, Tokyo, Japan) with 10, 20, or 40× objectives. A DC6 V digital camera (Olympus, Tokyo, Japan) coupled to the microscope was used to capture cell imaging. The morphology of cells in the presence or absence of treatments was compared.

#### 3.2.2. Fluorescence Microscopy

Fluorescence microscopy was used to confirm whether the compounds under study cross the cell membrane, and to understand where they are located within the cell (nucleus and/or cytoplasm). To this end, the procedure previously described in Lima et al. [39] was followed. Hoescht 33342 was used as a specific marker. Cells were seeded in 24-well culture plates containing sterile circular coverslips of 15 mm diameter. After 48 h for cell adhesion, they were incubated with the compounds for 20 h. After this incubation period, the cells were washed three times with PBS and the fixation process was carried out. To do this, the contents of the wells were removed, and a 4% formalin solution was added until the wells were covered, followed by an incubation at room temperature for 15 min. Then, the washing process was repeated three times with PBS, and the cells were then incubated with a solution of Hoescht 33342 (1:500) for 10 min, in the dark and at room temperature. Subsequently, the wells were washed again with PBS and the slides were mounted with permanent mounting medium (Dako) and left to dry in a cool and dry place for 24 h. Then, the cells were observed using the AxioImager Z2 microscope (Carl Zeiss Microscopy, White Plains, NY, USA) with the ZEN 2.6 PRO software and the Plan-Apochromat 40×/1.3 Oil DIC M27 objective (Carl Zeiss Microscopy, White Plains, NY, USA) coupled to two Axiocam 506 mono and Axiocam 503 color cameras (Carl Zeiss Microscopy, White Plains, NY, USA) and specifically using the Filter Set 38HE (Carl Zeiss Microscopy, White Plains, NY, USA).

#### 3.2.3. Flow Cytometry

Apoptosis (sub-G1 events) and cell cycle distribution were evaluated by propidium iodide (PI) staining and flow cytometry, as described by Riccardi and Nicolleti [20]. After 48 h of Caco-2 cells (2 mL; 3 × 10^4^ cells/mL) adherence in 6-well plates, the culture medium was substituted with the test solutions (3 mL and 5 mL for negative control) and incubated for a further 72 or 120 h. Untreated cells were used as the negative control, and sorbitol and 5-FU were used as positive controls. After incubation, the supernatant was collected in a centrifuge tube, cells were washed with PBS, trypsinized and wells were once again washed with PBS. This cell suspension was maintained on ice, centrifugated at 300 g for 5 min, and then the pellet was resuspended in 0.5 mL of PBS. Cells were fixed by adding 4.5 mL of 70% ethanol cooled at −20 °C on ice and were stored at −20 °C for a period ranging from one to a few days. On the day of flow cytometry analysis, PBS was added to the fixed cell tubes, followed by centrifugation and removal of the supernatant. The pellet was resuspended in 0.5 mL of PBS and 0.5 mL of DNA extraction solution (192 mL of 0.2 M sodium phosphate and 8 mL of 0.1% Triton X-100, pH 7.8). After incubation for 5 min, the cells were once again centrifuged, the supernatant was removed, and the pellet was resuspended in 0.3 mL of staining solution (20 µg/mL of PI and 0.2 mg/mL of DNase-free RNase in PBS). Using a FACSCalibur flow cytometer (Becton Dickinson, Franklin Lakes, NJ, USA) with a 488 nm laser line, a minimum of 20,000 events were acquired and recorded using the BD CellQuestTM Pro 4.0.2 software, and analyzed with FlowJo 10.5.3 software. To exclude debris and necrotic cells, events with a lower diameter (forward scatter; FCS) and PI fluorescence (FL3) than the hypodiploid apoptotic cells were eliminated (Figures S1 and S2). Two independent experiments were performed. A Student’s *t*-test was performed to evaluate the statistical significance of the results.

#### 3.2.4. Topoisomerase II Inhibitory Assay

Cyanine stock solutions in DMSO were further diluted with Tris-HCl (DMSO:Tris-HCl–1:9) to the concentrations desired for assays. The Topoisomerase II drug screening kit for 100 assays (Catalog No. 1009-1) was purchased from TopoGEN Inc. (Buena Vista, CO, USA). Experiments followed the manufacturer’s instructions. Briefly, all the ingredients (water, buffer, pRYG DNA supercoiled, test compound, and enzyme) were pooled together in a microcentrifuge tube on ice followed by 30 min of incubation at 37 °C. As suggested by the manufacturer, 5.0 units of enzyme and 250.0 ng of pRYG DNA were used. To stop the reactions, 2 µL of 10% SDS was added to the tubes at 37 °C. The tubes were further incubated at 37 °C for 15 min after the addition of Proteinase K 50 ug/mL (Thermo Fisher Scientific, Waltham, MA, USA), which was used to digest the enzyme. Then, the samples were loaded onto 1% agarose gel, prepared in Tris-Acetate-EDTA (TAE) buffer (40 mM Tris base, 20 mM acetic acid, 1 mM EDTA, pH 8.0) and stained with 0.012 µL/mL of GreenSafe (NZYTech, Lisbon, Portugal). Electrophoresis was run at 110 V for 40 min and analyzed under ultraviolet (UV) light using a FireReader Imaging System (UVITEC, Cambridge, UK). Band intensities were measured using the software Image Lab, Version 6.1 (BioRad Laboratories, Hercules, CA, USA). Experiments were repeated twice independently.

### 3.3. Computational Studies

The physicochemical properties, drug-likeness, and medicinal chemistry principles of dyes **5** and **10** were in silico-predicted by employing the SwissADME web tool [24].

## 4. Conclusions

A series of asymmetric monomethine cyanine dyes featuring benzothiazole, benzoxazole, benzimidazole, and quinoline heterocyclic rings with various substituents were successfully designed and synthesized with moderate to good yields. These compounds were then structurally characterized. Their cytotoxic effects were evaluated on human tumor cell lines for colorectal (Caco-2), breast (MCF-7), and prostate (PC-3) cancers, as well as on normal human dermal fibroblasts (NHDF), to assess their potential as antitumor agents. Notably, these dyes showed more potent and selective effects in colorectal cancer cells, especially in dyes derived from combinations like *N*-methylbenzoxazole and *N*-methylquinoline (dye **5**), and *N*-ethylbenzothiazole and *N*-ethyl-6-nitrobenzothiazole (dye **10**). The IC_50_ (SI) values for these dyes in Caco-2 cells were 10 nM (278) and 140 nM (151), respectively. Despite these promising IC_50_ and SI values, which are even better than those reported for the colorectal cancer agent 5-FU, it would be worthwhile to continue developing new derivatives with enhanced potency and selectivity, aiming to advance toward more effective potential anticancer agents.

Further in vitro studies to explore the mechanism of action revealed that both dyes readily enter cells and localize in the cytoplasm and nucleus. Dye **5** induced morphological changes suggestive of apoptosis, confirmed by flow cytometry, which showed a small but statistically significant increase in apoptotic cells compared to the negative control. While dye **5** did not have a significant effect on the cell cycle of Caco-2 cells, dye **10** caused a notable arrest in the G0/G1 phase.

Given that azacyanines have been previously reported as topoisomerase II inhibitors, we explored whether dyes **5** and **10** exhibited similar inhibitory properties. Remarkably, both compounds inhibited topoisomerase II at 50 µM, showing greater potency than the positive control (VP-16).

In silico studies predicted favorable physicochemical properties, drug-likeness, and pharmacokinetics for both dyes, suggesting good oral bioavailability. However, a potential toxicity warning was associated with the nitro aromatic group in dye **10**.

Considering all the data presented herein, it can be concluded that several of these asymmetric monomethine cyanine dyes hold promise as potential cancer therapeutics, particularly cyanine **5**. With its superior potency and selectivity, dye **5** stands out as a strong candidate for further investigation, including incorporation into suitable drug delivery systems and evaluation via in vivo studies for efficacy and safety, as a potential agent for colorectal cancer chemotherapy.

## Figures and Tables

**Table 1:** Structure, yield, maximum absorption wavelength, and logarithm of molar absorption coefficient (log ε) of dyes **1**–**12**.

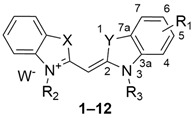								
Dye—Method	R_1_	R_2_	R_3_	X	Y	W	Yield (%)	λ_max_ (nm) (log ε)
**1** ^a^	H	CH_3_	C_5_H_11_	S	S	I	72	425 (4.85)
**2** ^a^	H	CH_3_	C_10_H_21_	S	S	I	68	427 (4.95)
**3** ^b^	H	CH_3_	CH_3_	S	O	TsO	47	401 (4.88)
**4** ^b^	H	CH_3_	CH_3_	S	NCH_3_	I	84	385 (5.26)
**5** ^b^	H	CH_3_	CH_3_	O	CH=CH	TsO	63	436 (4.72)
**6** ^b^	H	CH_3_	CH_3_	S	CH=CH	TsO	72	483 (4.53)
**7** ^b^	5-NHCOCH_3_	C_2_H_5_	C_2_H_5_	S	S	TsO	29	432 (4.92)
**8** ^c^	5-NH_2_	C_2_H_5_	C_2_H_5_	S	S	I	31	454 (4.67)
**9** ^b^	5-NO_2_	C_2_H_5_	C_2_H_5_	S	NCH_2_CH_3_	I	26	422 (4.56)
**10** ^b^	6-NO_2_	C_2_H_5_	C_2_H_5_	S	S	TsO	53	439 (4.81)
**11** ^d^	6-NH_2_	C_2_H_5_	C_2_H_5_	S	S	I	81	444 (4.62)
**12** ^e^	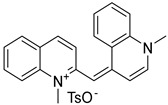						68	558 (5.26)

^a^ Thioalkyl method; ^b^ Thioalkyl method by one-pot synthesis; ^c^ Acid hydrolysis of **7**; ^d^ Reduction of **10**; ^e^ Potassium hydroxide method.

**Table 2:** Calculated IC_50_ values, respective 95% confidence intervals (CI), and selectivity index (SI) for cancer versus non-cancer cell line NHDF of cyanine dyes **1**–**12**, and control 5-FU on NHDF, Caco-2, PC-3, and MCF-7 cell lines after 72 h of incubation. Results are referent to at least two independent assays performed in quadruplicate.

Dye	NHDF		Caco-2			PC-3			MCF-7		
	IC_50_	95% CI	IC_50_	95% CI	SI	IC_50_	95% CI	SI	IC_50_	95% CI	SI
5-FU	4.53	2.20–9.32	1.11	0.66–1.89	4.1	2.43	1.10–5.38	1.9	2.12	1.22–3.70	2.1
**1**	0.55	0.42–0.70	0.36	0.28–0.46	1.5	-	-	-	-	-	-
**2**	0.27	0.19–0.39	0.24	0.17–0.35	1.1	-	-	-	-	-	-
**3**	7.53	4.09–13.84	0.09	0.06–0.14	81.2	0.43	0.28–0.67	17.2	4.04	1.78–9.15	1.9
**4**	7.93	4.44–14.14	0.23	0.15–0.35	33.6	2.85	1.35–6.01	2.8	-	-	-
**5**	2.79	1.68–4.65	0.010	0.006–0.016	278.3	1.43	0.99–2.09	1.9	3.83	1.74–8.44	0.7
**6**	0.79	0.59–1.05	0.27	0.14–0.54	2.9	-	-	-	-	-	-
**7**	11.63	7.11–19.0	0.14	0.10–0.19	80.5	3.56	1.55–8.16	3.3	0.38	0.10–1.38	30.2
**8**	0.95	0.77–1.17	0.74	0.44–1.20	1.3	-	-	-	-	-	-
**9**	24.54	11.16–53.96	1.40	0.86–2.28	17.5	-	-	-	-	-	-
**10**	22.38	10.47–47-85	0.14	0.08–0.25	151.4	5.93	2.65–13.28	3.8	13.30	6.84–25.86	1.7
**11**	2.997	1.82–4.92	0.17	0.13–0.23	17.1	2.10	1.30–3.38	1.4	0.33	0.16–0.65	9.0
**12**	0.63	0.43–0.89	0.73	0.58–0.96	0.9	-	-	-	-	-	-

**Table 3:** In silico-predicted physicochemical properties, drug-likeness, and medicinal chemistry principles of dyes **5** and **10**.

Descriptor		Dye 5	Dye 10
Physicochemical Properties	H-bond donors	0	0
	H-bond acceptors	1	2
	Molecular weight (g/mol)	289.35	384.50
	Consensus CLogP	2.74	3.36
	Molar Refractivity	90.60	112.53
	Rotatable bonds	1	4
	Fraction Csp^3^	0.11	0.21
	TPSA (Å^2^)	21.95	111.11
	Log S (ESOL)	−5.07	−5.94
Drug-likeness	Lipinski	Yes; 0 violations	Yes; 0 violations
	Ghose	Yes	Yes
	Veber	Yes	Yes
	Egan	Yes	Yes
	Muegge	Yes	Yes
	Bioavailability Score	0.55	0.55
Medicinal Chemistry	PAINS	0 alert	0 alert
	Brenk	1 alert; Quaternary nitrogen	2 alerts; Quaternary nitrogen; nitro group
	Leadlikeness	Yes	No; 1 violation; MW > 350
	Synthetic accessibility	3.56	3.95

## Data Availability

The original contributions presented in this study are partially presented in the Supplementary Materials. Remaining data are available upon request from the corresponding authors.

## Supplementary Materials

The following supporting information can be downloaded at: https://www.mdpi.com/article/10.3390/molecules29235581/s1, Table S1: Characteristic ^1^H and ^13^C NMR spectra signal of dyes **1**–**12**; Table S2: Data for in vitro effects of all studied compounds on cell viability of PC-3, MCF-7, Caco-2, and NHDF cell lines, at a single concentration of 10 µM; Figures S1 and S2: Representative plots and histograms of flow cytometry for the apoptosis induction and cell cycle effects analysis; Figures S3–S14: ^1^H and ^13^C NMR spectra for cyanine dyes **1**–**12**; Figures S15–S20: ESI-HRMS spectra for cyanine dyes **1**, **2** and **7**–**10**.
