# Supplementary material for: A New Demand for Improved Selectivity and Potency of Cyanine Dyes as Antiproliferative Agents Against Colorectal Cancer Cells
Source: Molecules. 2024 Nov 26;29(23):5581. doi: 10.3390/molecules29235581 (PMC11642940; doi:10.3390/molecules29235581)
Supplement: Supplementary file 1 [file molecules-29-05581-s001.zip › molecules-3265966-supplementary.pdf]

molecules

MDPI

Article

A New Demand for Improved Selectivity and Potency of Cyanine Dyes as Antiproliferative Agents Against Colorectal Cancer Cells

Ana Maia [1,2,†], Cathy Ventura [1], Adriana O. Santos [1], Maria J. Nunes [2], Renato E. F. Boto [1,2], Ângela Sousa [1], Samuel M. Silvestre [1,2], Paulo Almeida [1,2,*] and João L. Serrano [1,2,*,†]

[1] CICS-UBI—Health Sciences Research Center, University of Beira Interior, Av. Infante D. Henrique, 6201-506 Covilhã, Portugal; anaamaia97@hotmail.com (A.M.); cathy.ventura@ubi.pt (C.V.); asantos@fcsaude.ubi.pt (A.O.S.); rboto@ubi.pt (R.E.F.B.); angela@fcsaude.ubi.pt (Â.S.); sms@ubi.pt (S.M.S.)

[2] Department of Chemistry, University of Beira Interior, Rua Marquês de Ávila e Bolama, 6201-001 Covilhã, Portugal; mjnunes@ubi.pt

* Correspondence: paulo.almeida@ubi.pt (P.A.); joao.serrano@ubi.pt (J.L.S.)

† These authors contributed equally to this work.

Table of Contents

Table S1 – Characteristic ^{1}H and ^{13}C NMR spectra signal of dyes 1-12. 3

Table S2 – Data for in vitro effects of dyes **1**-**12** and 5-fluorouracil (5-FU) on cell viability of non-tumor cell line of normal human dermal fibroblasts (NHDF) and human adenocarcinoma cell lines of the colorectal (Caco-2), prostate (PC-3) and breast (MCF-7), after 72 h of incubation at a single concentration of 10 μM.[a] 4

Figure S1 – Representative flow cytometry plots used for debris exclusion and single cells selections in the analysis of subG1 events (apoptosis) and cell cycle effect histograms of dyes **5** (1 μM) and **10** (10 μM), as well as the positive controls, sorbitol (600 mM) and 5-FU (10 μM), on human colorectal adenocarcinoma Caco-2 cell line after 72 hours of treatment. Sorbitol was analyzed after the last 24 hours of incubation. Data is representative of one of the carried-out experiments... 6

Figure S2 – Representative flow cytometry plots used for debris exclusion and single cells selections in the analysis of subG1 events (apoptosis) and cell cycle effect histograms of dyes **5** (1 μM) and **10** (10 μM), as well as the positive controls, sorbitol (600 mM) and 5-FU (10 μM), on human colorectal adenocarcinoma Caco-2 cell line after 72 hours of treatment. Sorbitol was analyzed after the last 24 hours of incubation. Data are representative of one of the carried-out experiments... 8

Figure S3 – ^{1}H and ^{13}C NMR spectra of cyanine dye **1**... 10

Figure S4 – ^{1}H and ^{13}C NMR spectra of cyanine dye **2**... 11

Figure S5 – ^{1}H and ^{13}C NMR spectra of cyanine dye **3**... 12

Figure S6 – ^{1}H and ^{13}C NMR spectra of cyanine dye **4**... 13

Figure S7 – ^{1}H and ^{13}C NMR spectra of cyanine dye **5**... 14

Figure S8 – ^{1}H and ^{13}C NMR spectra of cyanine dye **6**... 15

Figure S9 – ^{1}H and ^{13}C NMR spectra of cyanine dye **7**... 16

Figure S10 – ^{1}H and ^{13}C NMR spectra of cyanine dye **8**... 17

Figure S11 – ^{1}H and ^{13}C NMR spectra of cyanine dye **9**... 18

Figure S12 – ^{1}H and ^{13}C NMR spectra of cyanine dye **10**... 19

Figure S13 – ^{1}H and ^{13}C NMR spectra of cyanine dye **11**... 20

Figure S14 – ^{1}H and ^{13}C NMR spectra of cyanine dye **12**... 22

Figure S15 –HRMS spectrum of cyanine dye **1**. 22

Figure S16 –HRMS spectrum of cyanine dye **2**. 22

Figure S17 –HRMS spectrum of cyanine dye **7**. 23

Figure S18 –HRMS spectrum of cyanine dye **8**. 23

Figure S19 –HRMS spectrum of cyanine dye **9**. 24

Figure S20 –HRMS spectrum of cyanine dye **10**. 24

Table S1 – Characteristic ^{1}H and ^{13}C NMR spectra signal of dyes **1-12**.

Dye	^{1}H NMR		^{13}C NMR	
	C=C<u>H</u>	$N^{1/2+}C\underline{H}_n$	C=C<u>H</u>	$N^{1/2+}C\underline{H}_n$
**1**	6.70 (s, 1H)	4,62 (t, *J* = 7.5 Hz, 2H)<br>4.03 (s, 3H)	82.6	46.2<br>34.3
**2**	6.70 (s, 1H)	4,63 (t, *J* = 7.4 Hz, 2H)<br>4.02 (s, 3H)	82.7	46.2<br>34.2
**3**	6.28 (s, 1H)	3,98 (s, 3H)<br>3,84 (s, 3H)	69.8	34.0<br>30.6
**4**	5.79 (s, 1H)	3,90 (s, 6H)<br>3,70 (s, 3H)	68.5	33.3<br>32.8
**5**	5.62 (s, 1H)	4.10 (s, 3H)<br>3.83 (s, 3H)	72.4	37.5<br>30.6
**6**	6.16 (s, 1H)	4.12 (s, 3H)<br>3.94 (s, 3H)	86.5	37.6<br>33.9
**7**	6.74 (s, 1H)	4,69 (t, *J* = 7.2 Hz, 2H)<br>4,58 (t, *J* = 7.2 Hz, 2H)	82.1	41.7<br>41.6
**8**	6.69 (s, 1H)	4,66 (q, *J* = 7.1 Hz, 2H)<br>4,53 (t, *J* = 7.4 Hz, 2H)	81.6	41.5<br>41.3
**9**	5.88 (s, 1H)	4.59 (q, *J* = 7.1 Hz, 2H)<br>4.52 (q, *J* = 7.2 Hz, 2H)	67.6	42.2<br>42.1
**10**	6.85 (s, 1H)	4.77 (q, *J* = 7.5 Hz, 2H)<br>4.71 (q, *J* = 7.5 Hz, 2H)	83.9	42.2<br>42.0
**11**	6.54 (s, 1H)	4.57 (q, *J* = 7.4 Hz, 4H)	81.1	41.7<br>41.1
**12**	6.49 (s, 1H)	4.07 (s, 3H)<br>4.01 (s, 3H)	94.4	41.7<br>37.7

Table S2 – Data for in vitro effects of dyes **1-12** and 5-fluorouracil (5-FU) on cell viability of non-tumor cell line of normal human dermal fibroblasts (NHDF) and human adenocarcinoma cell lines of the colorectal (Caco-2), prostate (PC-3) and breast (MCF-7), after 72 h of incubation at a single concentration of 10 μM.[a]

	NHDF	Caco-2	PC-3	MCF-7
Control	100 ± 5.45	100 ± 6.69	100 ± 5.19	100 ± 3.14
5-FU	10.5 ± 0.74	30.12 ± 1.24	20.52 ± 2.3	25.29 ± 4.94
**1**	0.84 ± 0.26	1.37 ± 0.08	0.56 ± 0.12	0.87 ± 1.05
**2**	0.13 ± 0.43	1.09 ± 0.47	0.62 ± 0.1	0.62 ± 0.79
**3**	23.85 ± 3.05	5.81 ± 0.8	1.81 ± 0.31	3.04 ± 1.08
**4**	23.06 ± 1.91	7.15 ± 1.08	4.09 ± 0.61	21.65 ± 1.33
**5**	15.42 ± 1.82	5.05 ± 0.19	3.47 ± 0.95	6.34 ± 1.06
**6**	4.28 ± 1.24	1.93 ± 0.78	4.98 ± 1.62	2.49 ± 0.32
**7**	28.98 ± 4.76	9.89 ± 1.38	7.97 ± 4	11.76 ± 4.3
**8**	5.52 ± 0.69	5.21 ± 0.89	4.12 ± 1.71	6.18 ± 1.48
**9**	38.22 ± 4.27	16.42 ± 0.81	39.54 ± 7.97	52.94 ± 8.99
**10**	34.29 ± 1.99	18.61 ± 0.65	14.41 ± 1.6	10.59 ± 6.44
**11**	16.08 ± 2.46	9.6 ± 1.47	10.52 ± 4.24	7.65 ± 2.8
**12**	5.72 ± 1.91	10.74 ± 0.43	9.02 ± 3.29	4.81 ± 1.56

[a] Results are expressed as average values of cell viability percentage in relation to control ± standard deviation of two independent assays performed in quadruplicate. A $p < 0.001$ versus negative control in the statistical significance analysis (Student's t-test) was observed for all compounds.

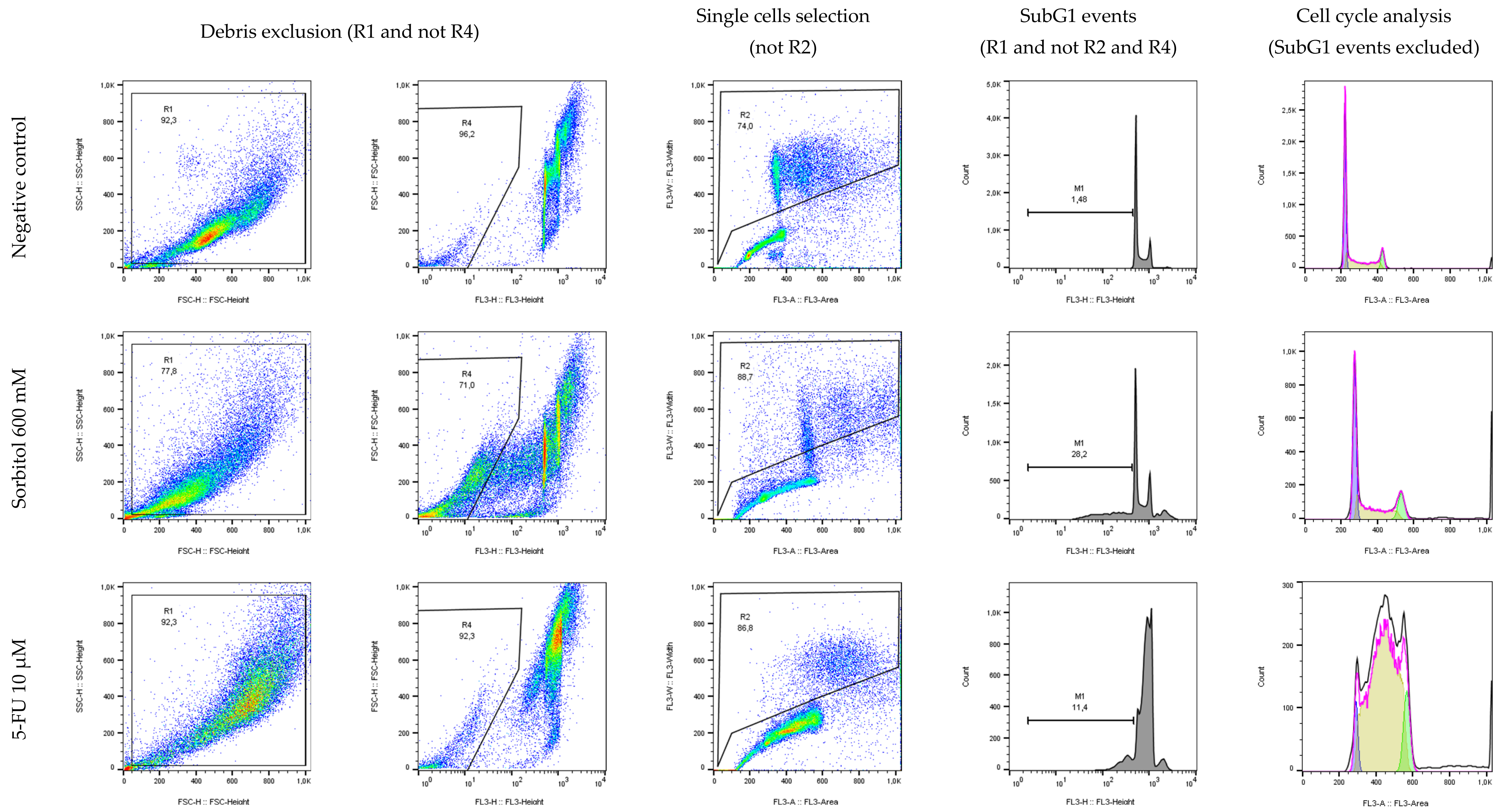
Debris exclusion (R1 and not R4)
Single cells selection (not R2)
SubG1 events (R1 and not R2 and R4)
Cell cycle analysis (SubG1 events excluded)
Negative control
Sorbitol 600 mM
5-FU 10 µM
R1 92,3
R4 96,2
R2 74,0
M1 1,48
R1 77,8
R4 71,0
R2 88,7
M1 28,2
R1 92,3
R4 92,3
R2 86,8
M1 11,4
SSC-H :: SSC-Height
FSC-H :: FSC-Height
FL3-H :: FL3-Height
FL3-W :: FL3-Width
FL3-A :: FL3-Area
Count

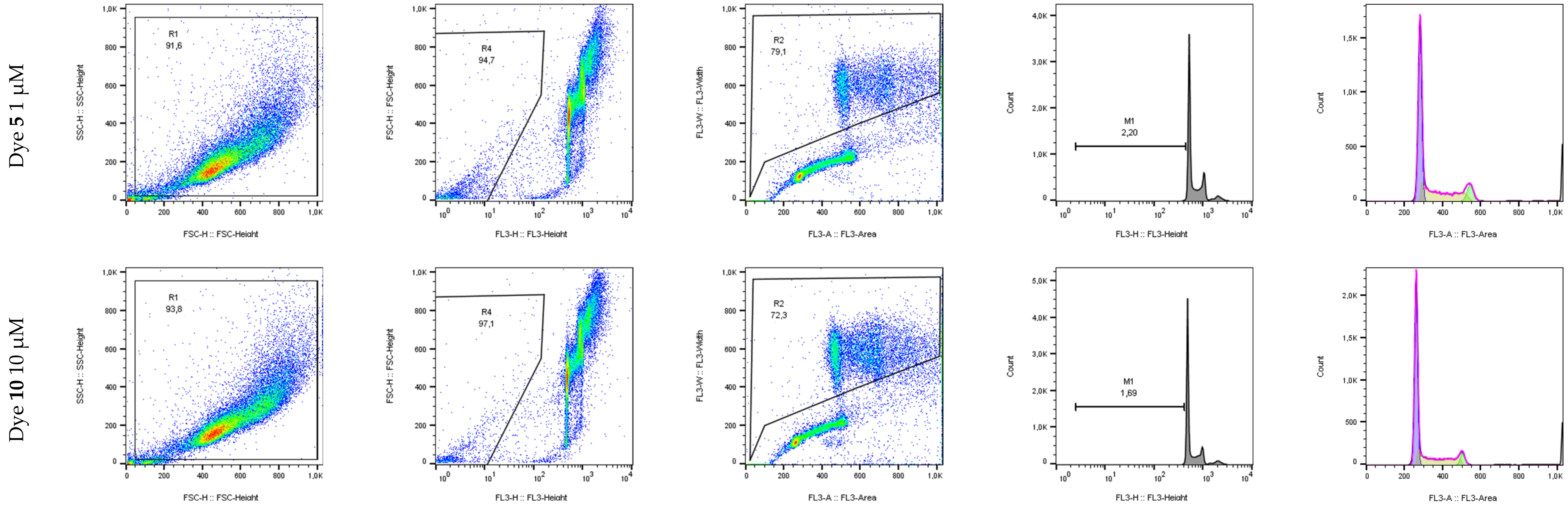

1.1. Figure S1 – Representative flow cytometry plots used for debris exclusion and single cells selections in the analysis of subG1 events (apoptosis) and cell cycle effect histograms of dyes 5 (1 µM) and 10 (10 µM), as well as the positive controls, sorbitol (600 mM) and 5-FU (10 µM), on human colorectal adenocarcinoma Caco-2 cell line after 72 hours of treatment. Sorbitol was analyzed after the last 24 hours of incubation. Data are representative of one of the experiments carried out.

Debris exclusion (R1 and not R4)	Single cells selection (not R2)	SubG1 events (R1 and not R2 and R4)	Cell cycle analysis (SubG1 events excluded)

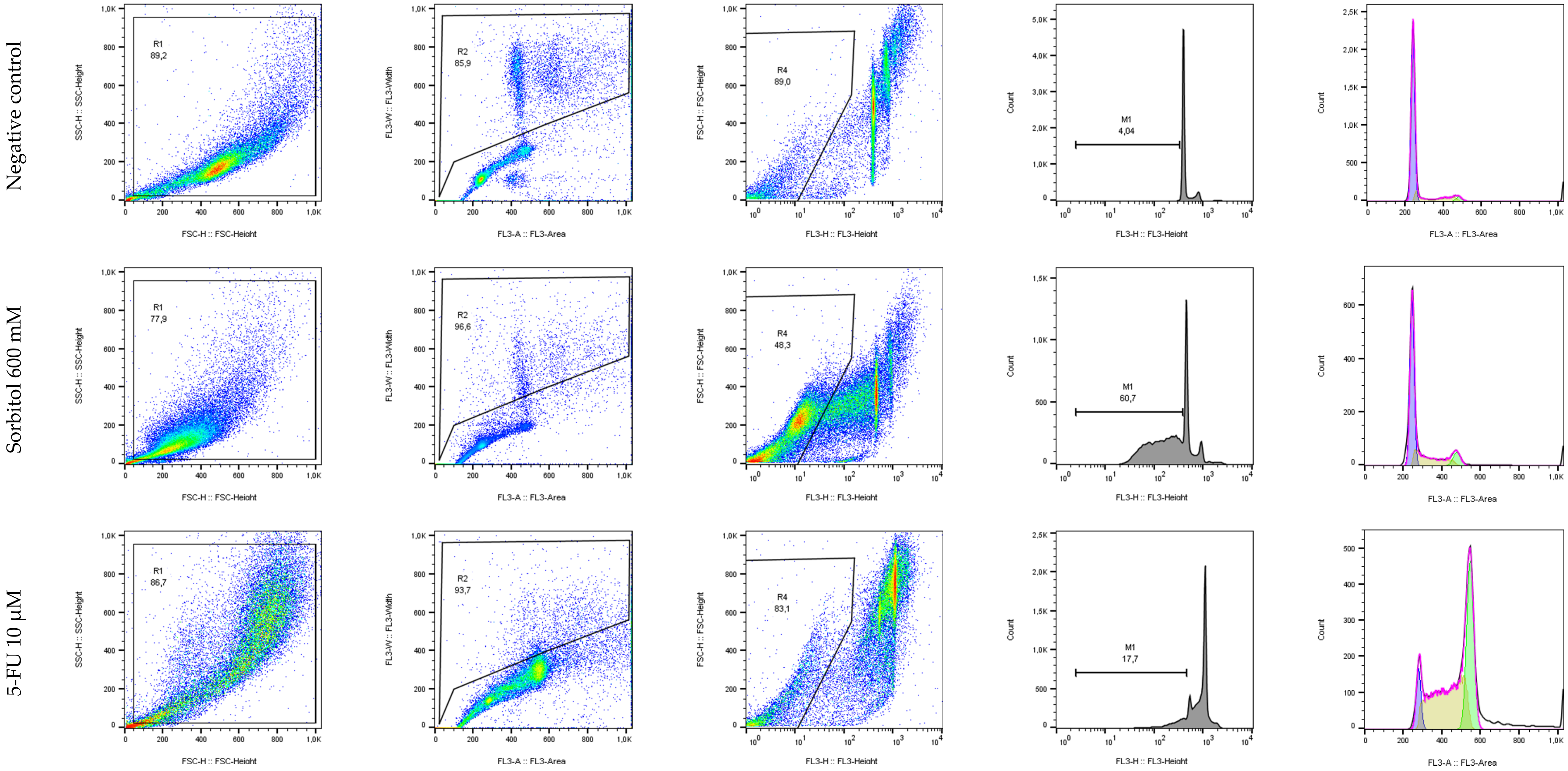
Negative control
Sorbitol 600 mM
5-FU 10 μM
R1 89,2
R2 85,9
R4 89,0
M1 4,04
R1 77,9
R2 96,6
R4 48,3
M1 60,7
R1 86,7
R2 93,7
R4 83,1
M1 17,7
SSC-H :: SSC-Height
FSC-H :: FSC-Height
FL3-W :: FL3-Width
FL3-A :: FL3-Area
FL3-H :: FL3-Height
Count

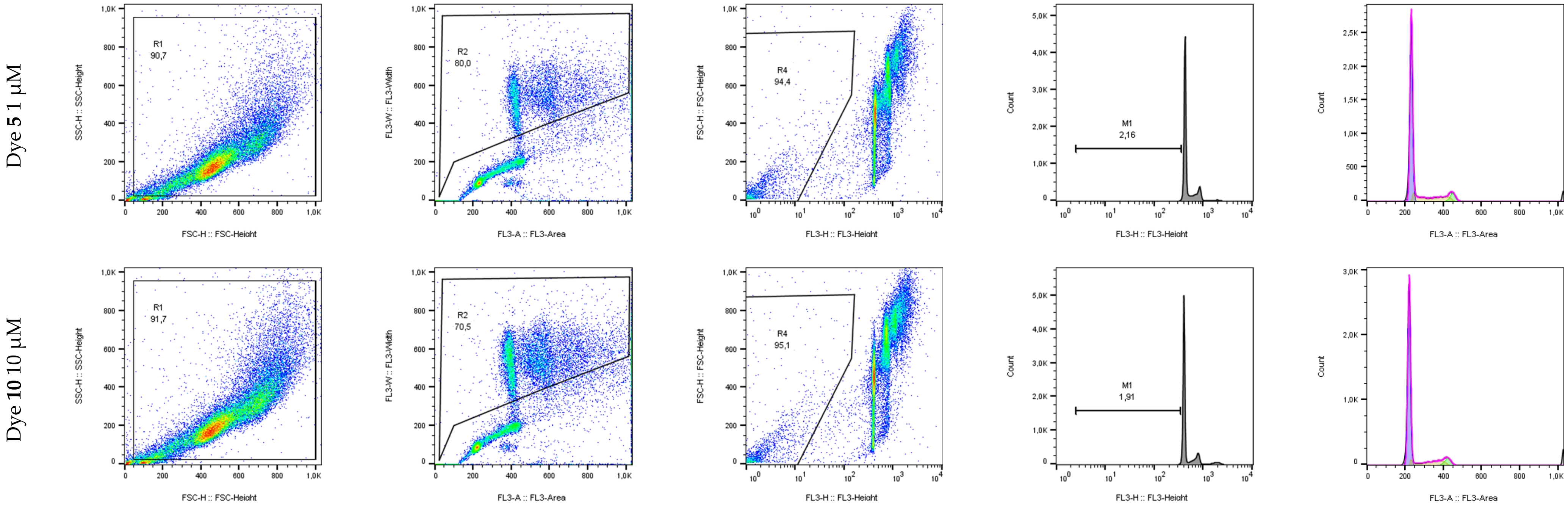

1.2. Figure S2 – Representative flow cytometry plots used for debris exclusion and single cells selections in the analysis of subG1 events (apoptosis) and cell cycle effect histograms of dyes 5 (1 μM) and 10 (10 μM), as well as the positive controls, sorbitol (600 mM) and 5-FU (10 μM), on human colorectal adenocarcinoma Caco-2 cell line after 72 hours of treatment. Sorbitol was analyzed after the last 24 hours of incubation. Data is representative of one of the experiments carried out.

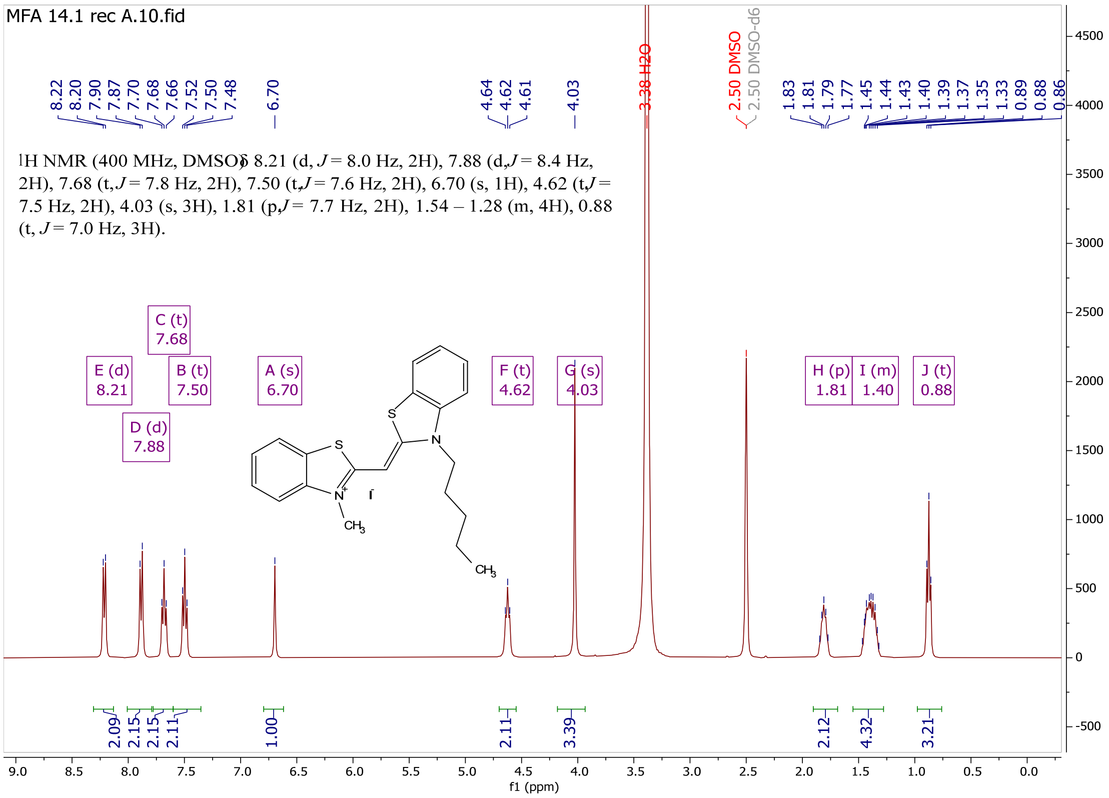

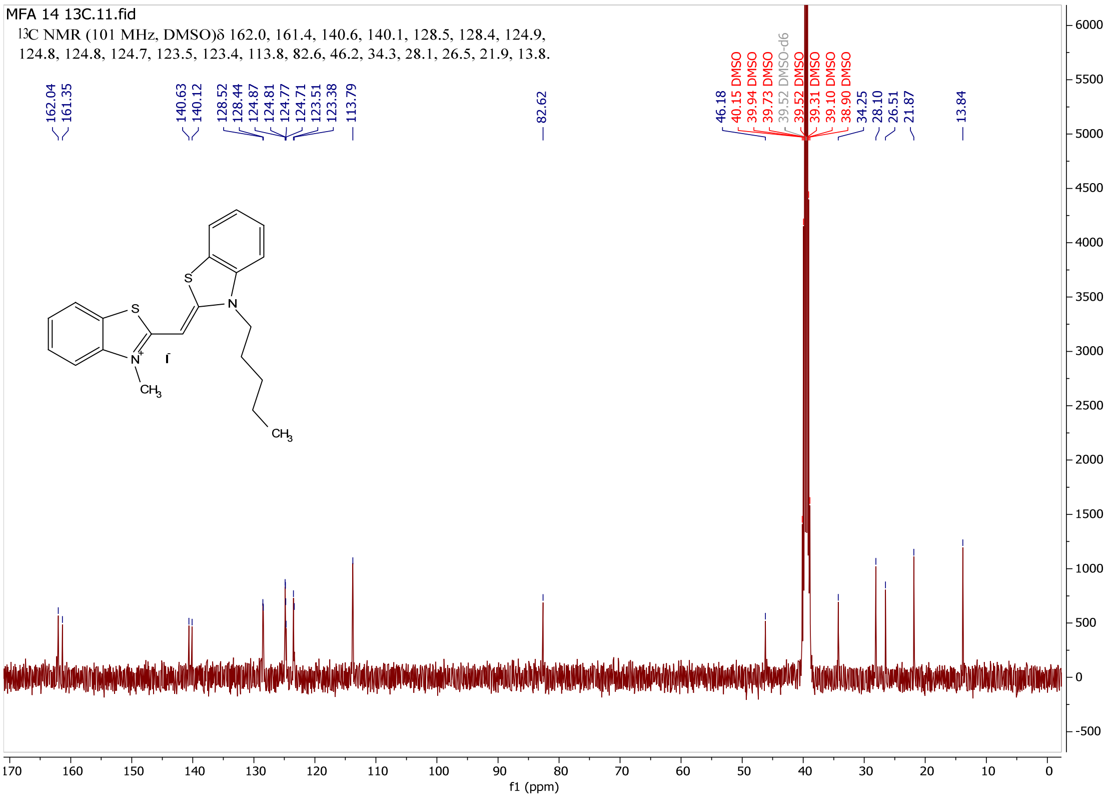

1.3. Figure S3 – ^{1}H and ^{13}C NMR spectra of cyanine dye 1.

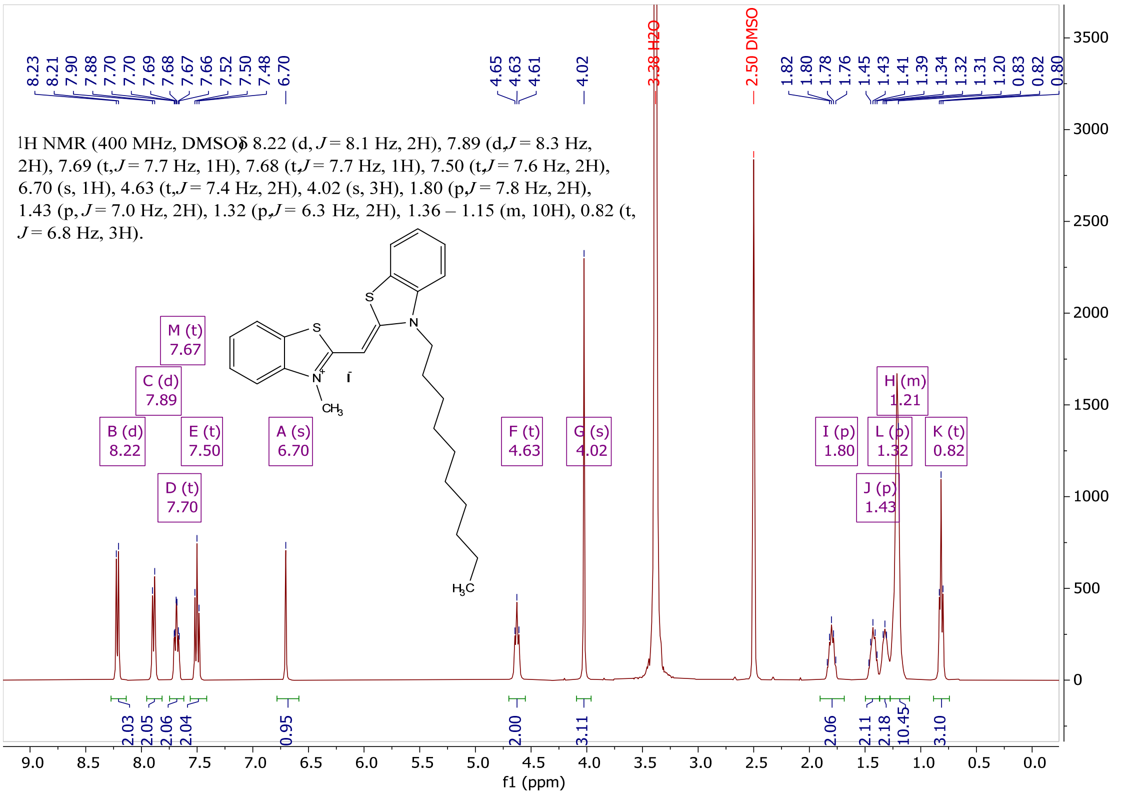

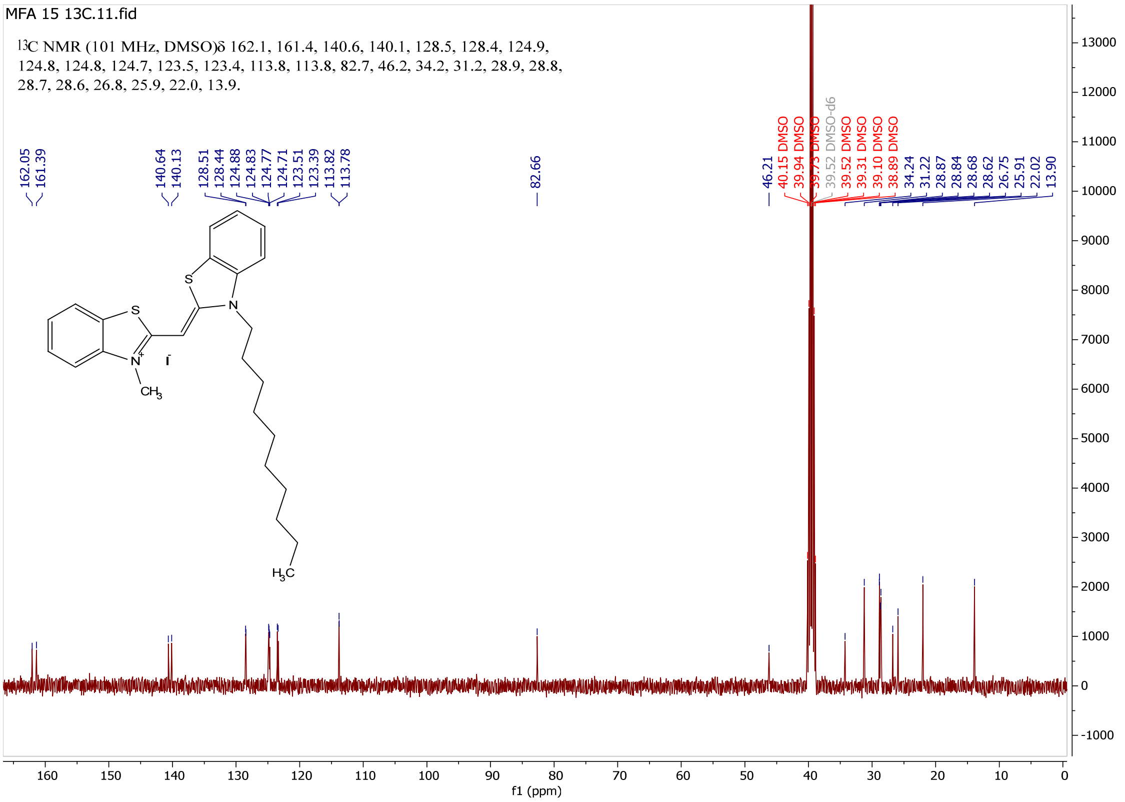

1.4. Figure S4 – ^{1}H and ^{13}C NMR spectra of cyanine dye 2.

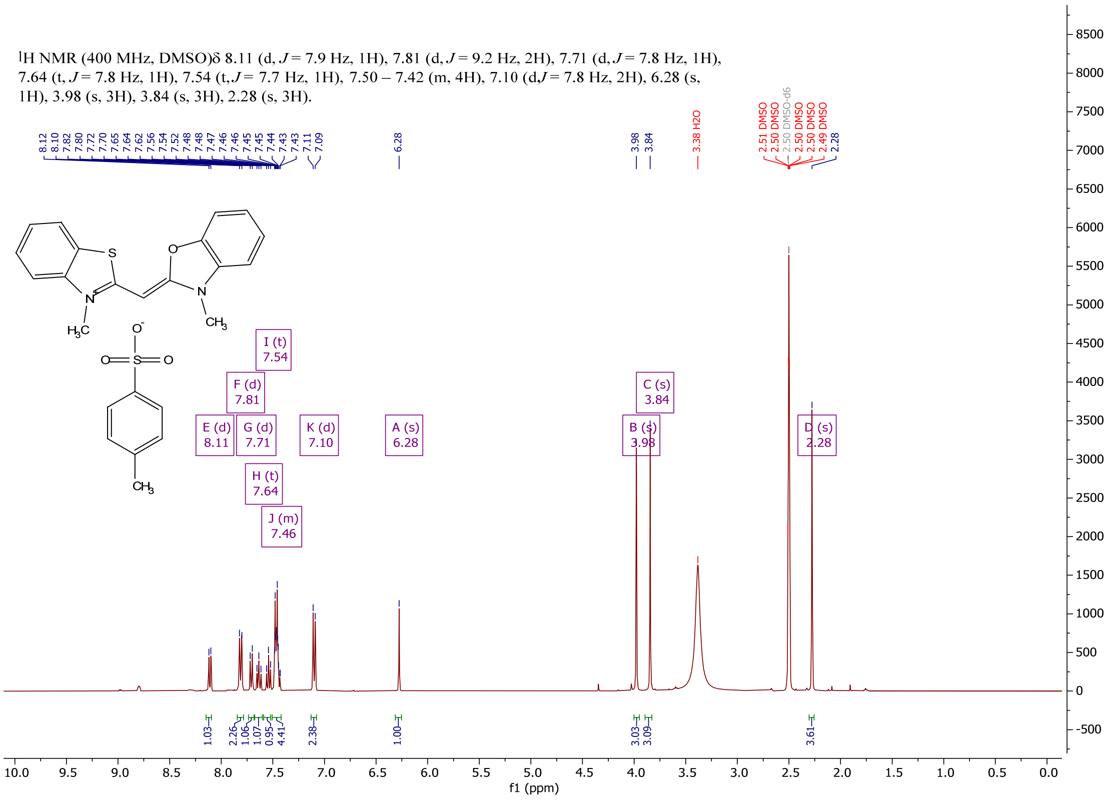

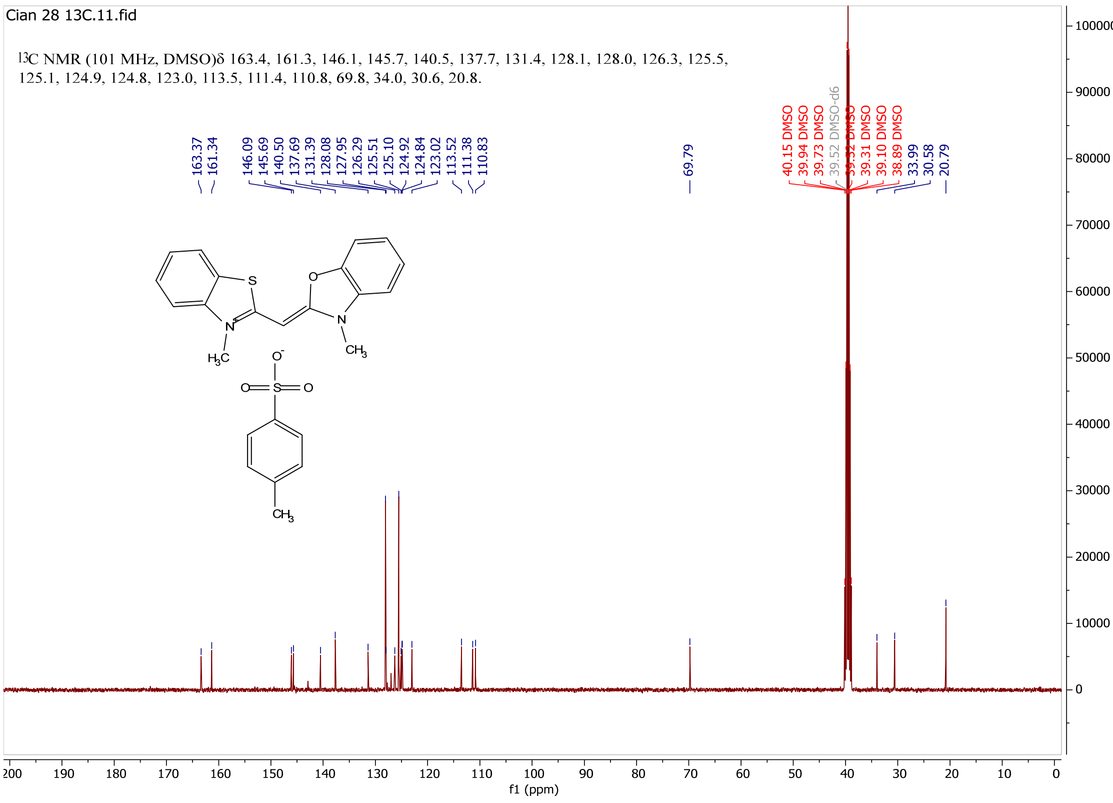

1.5. Figure S5 – ^{1}H and ^{13}C NMR spectra of cyanine dye 3.

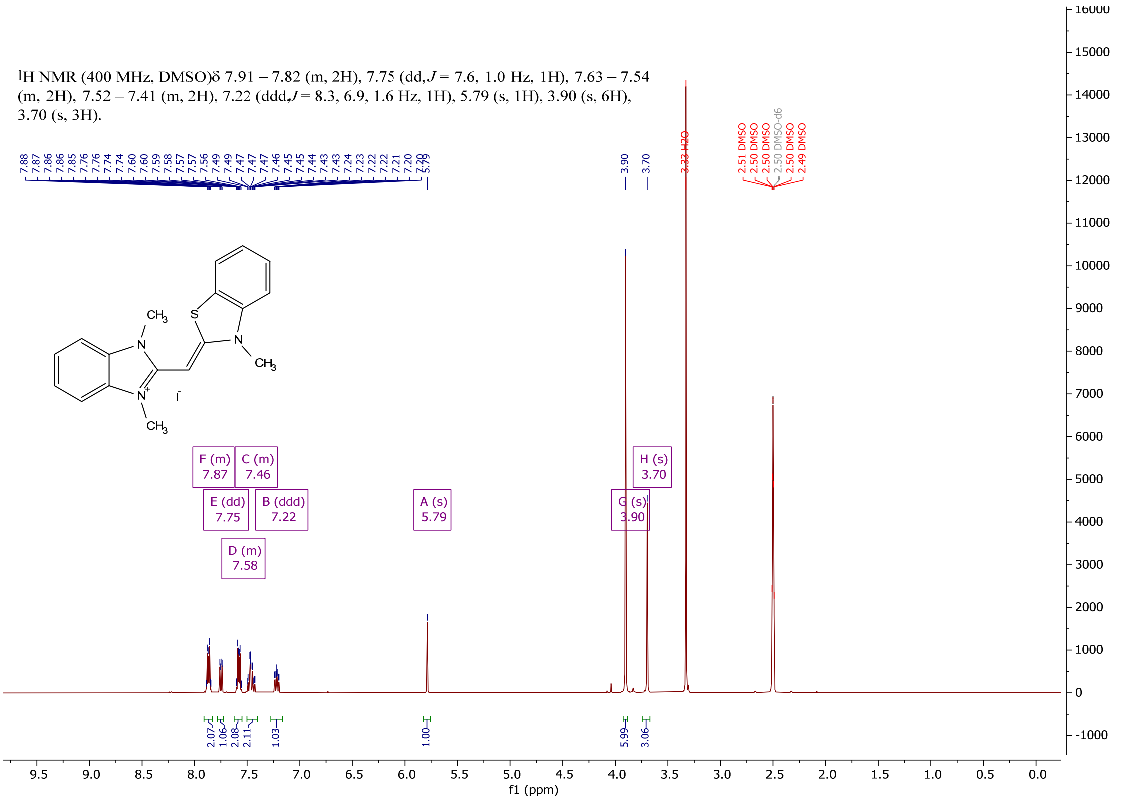

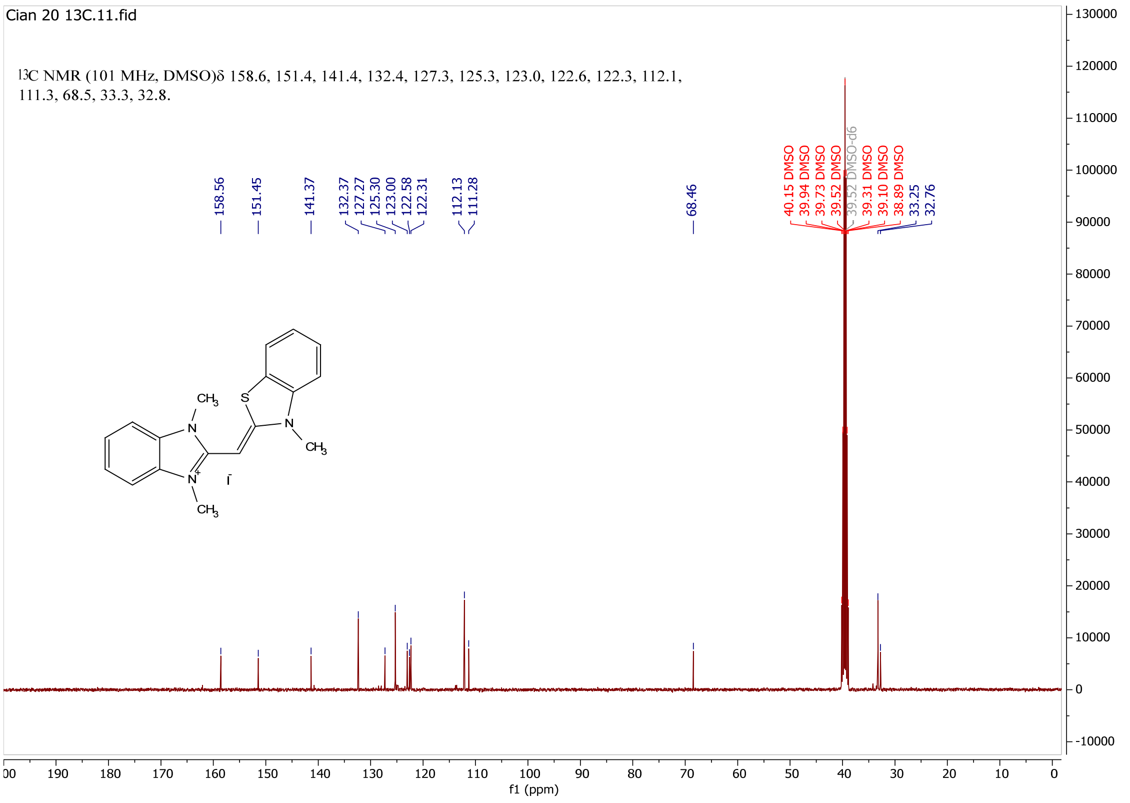

1.6. Figure S6 – ^{1}H and ^{13}C NMR spectra of cyanine dye 4.

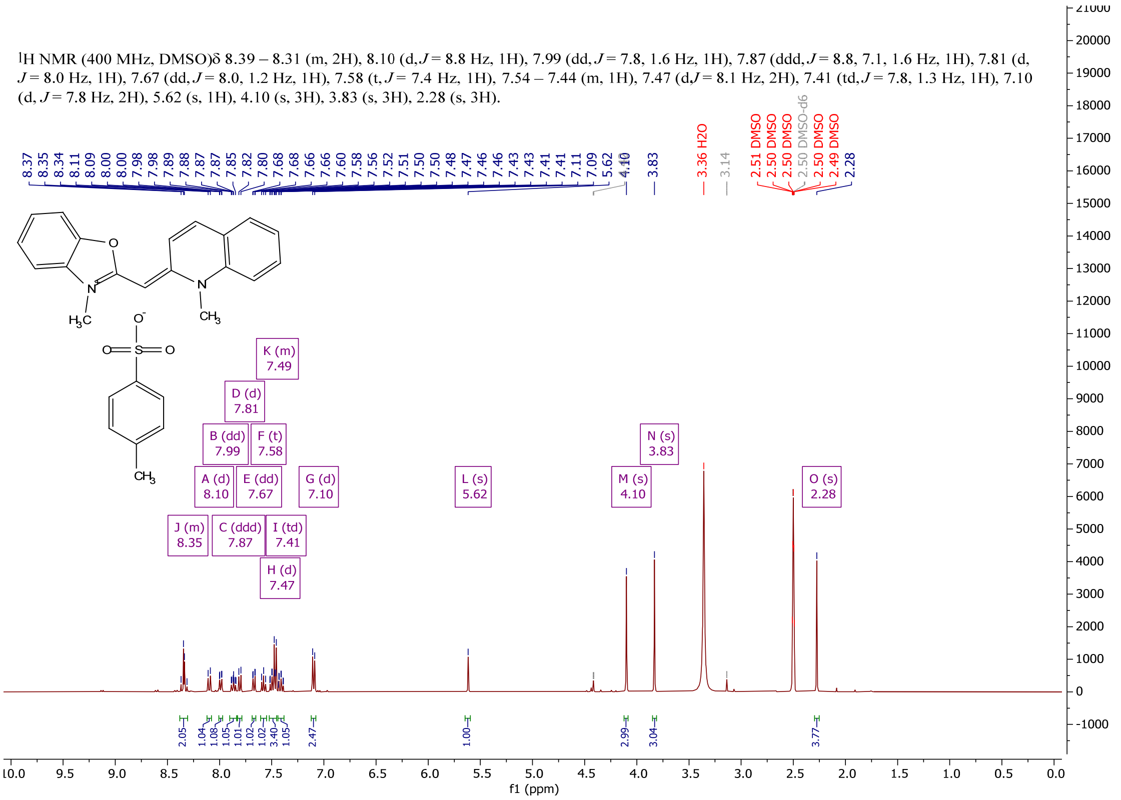

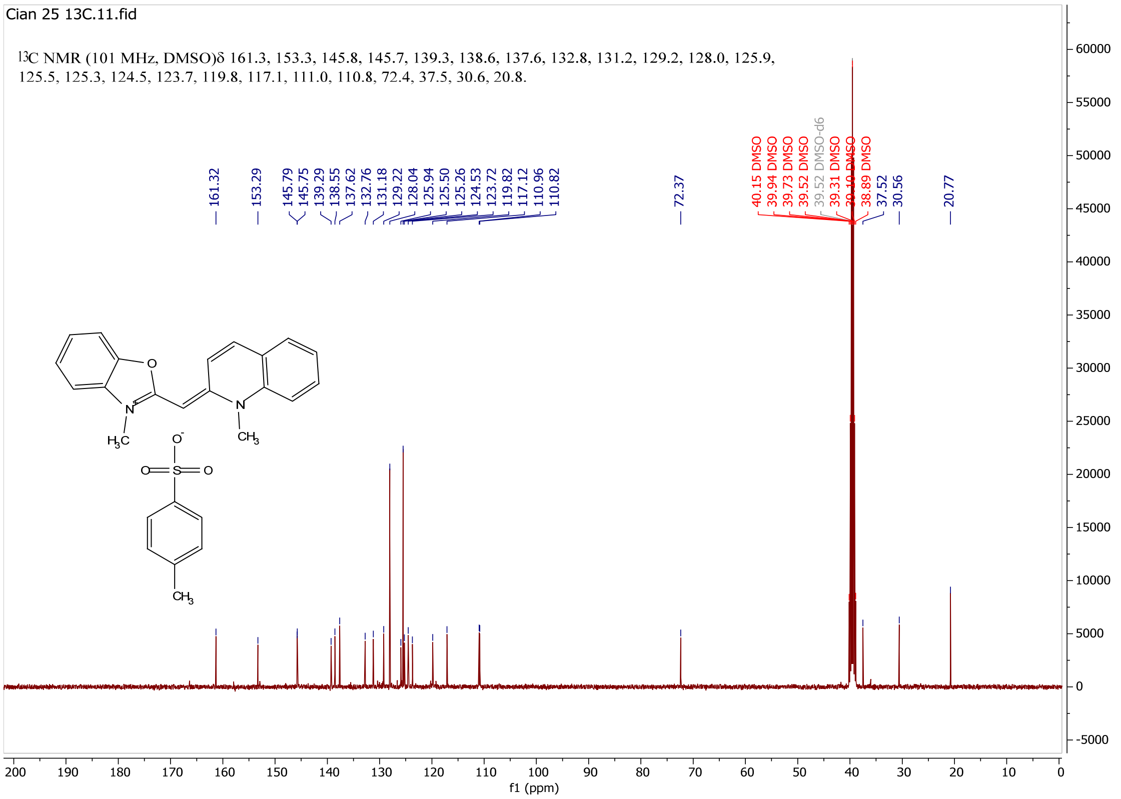

1.7. Figure S7 – ^{1}H and ^{13}C NMR spectra of cyanine dye 5.

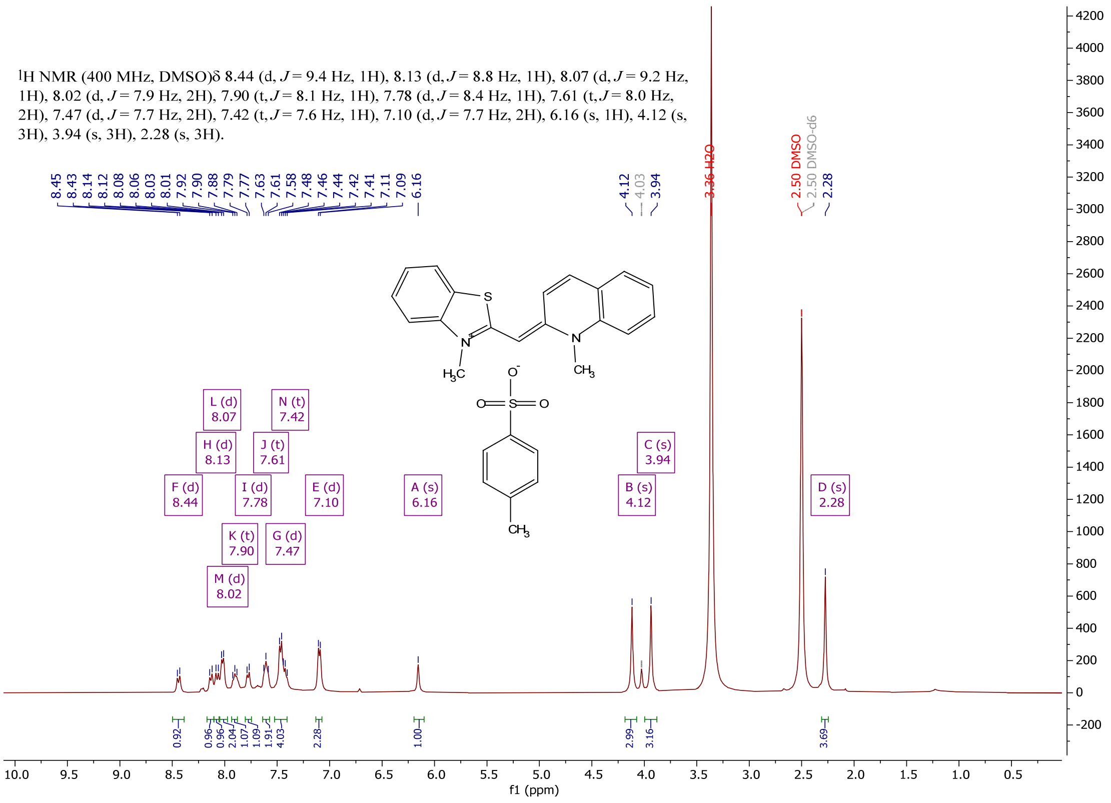

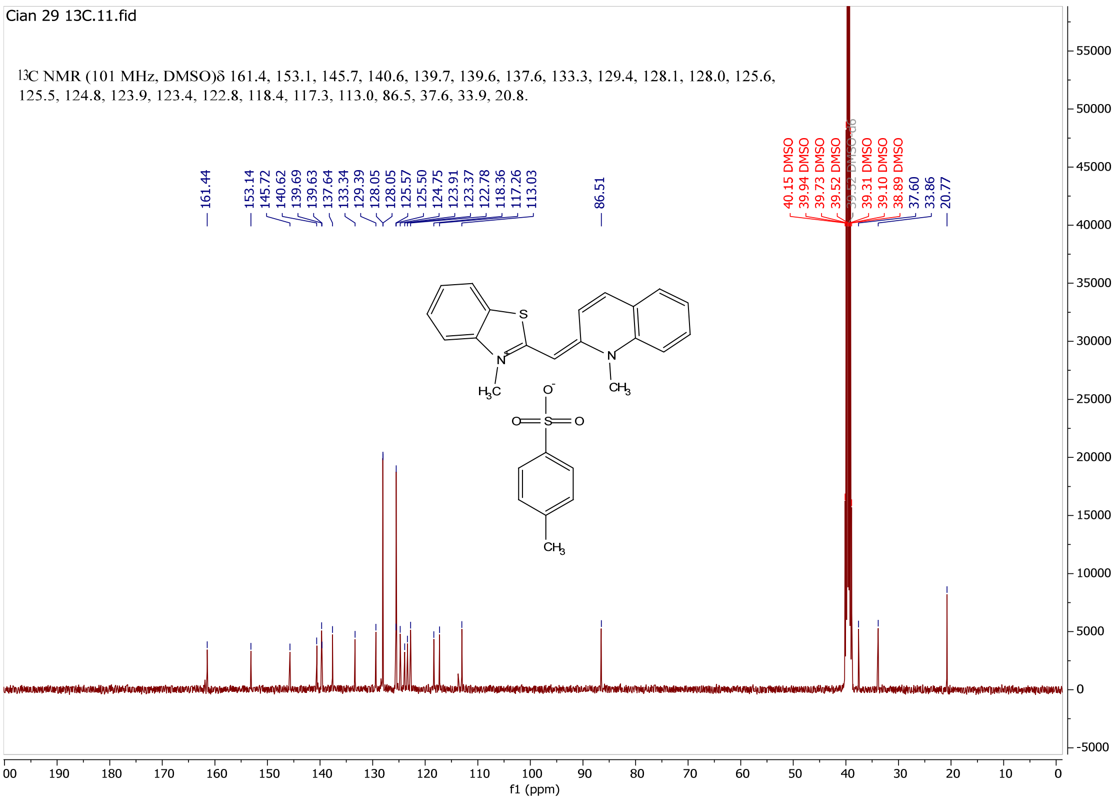

1.8. Figure S8 – ^{1}H and ^{13}C NMR spectra of cyanine dye 6.

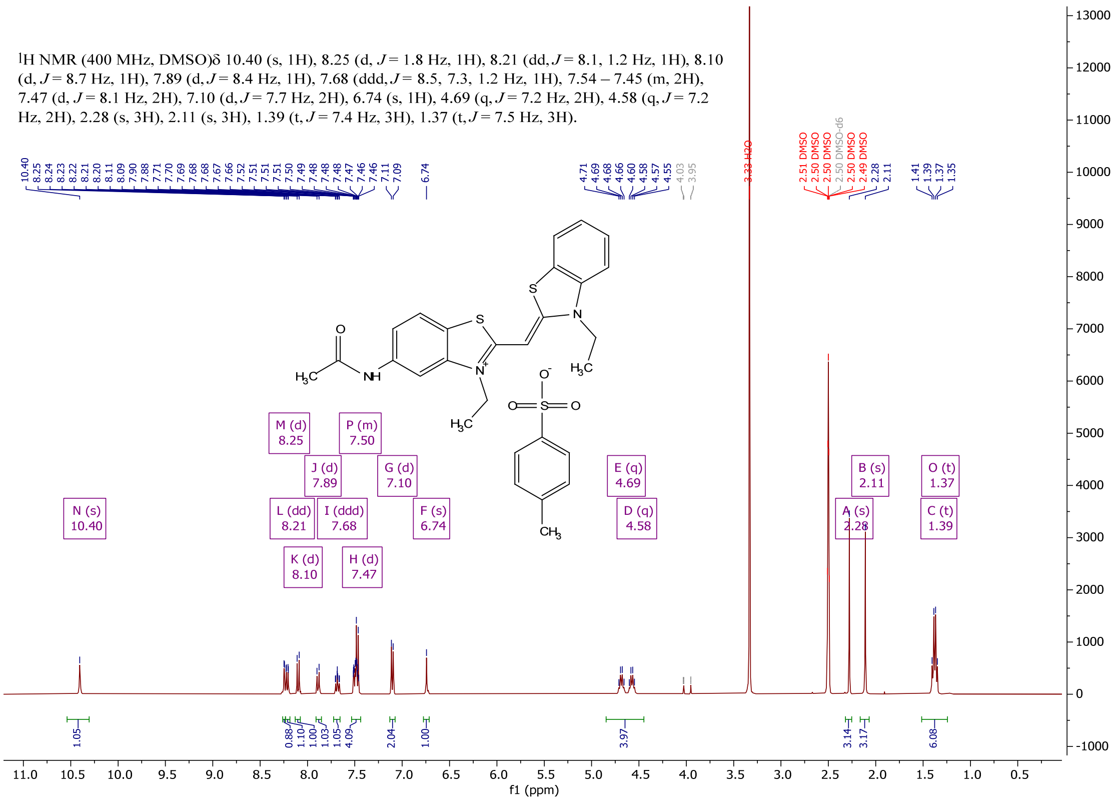

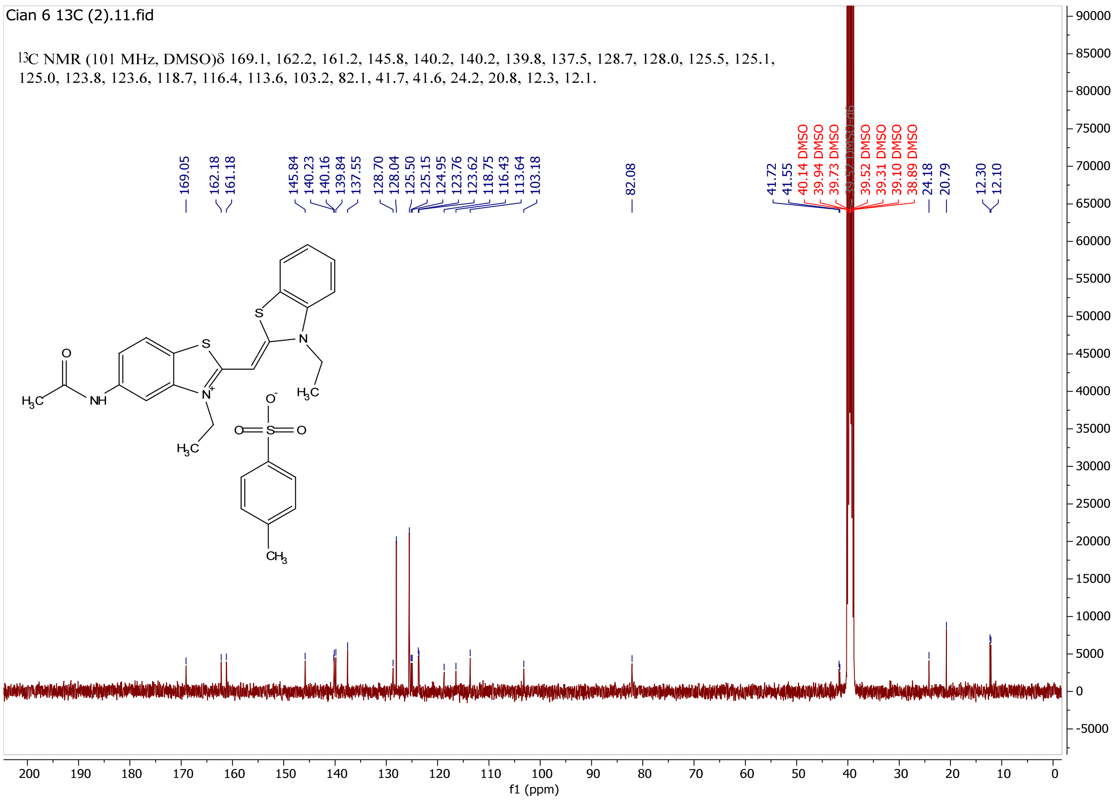

1.9. Figure S9 – 1H and 13C NMR spectra of cyanine dye 7.

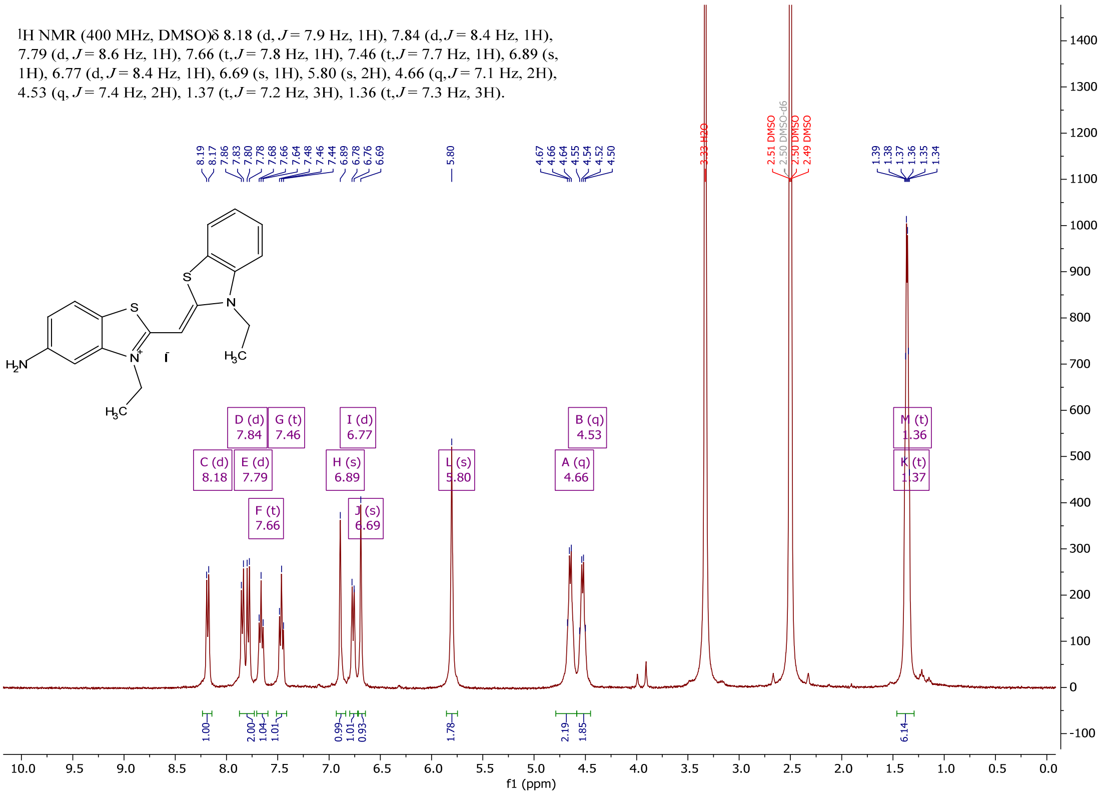

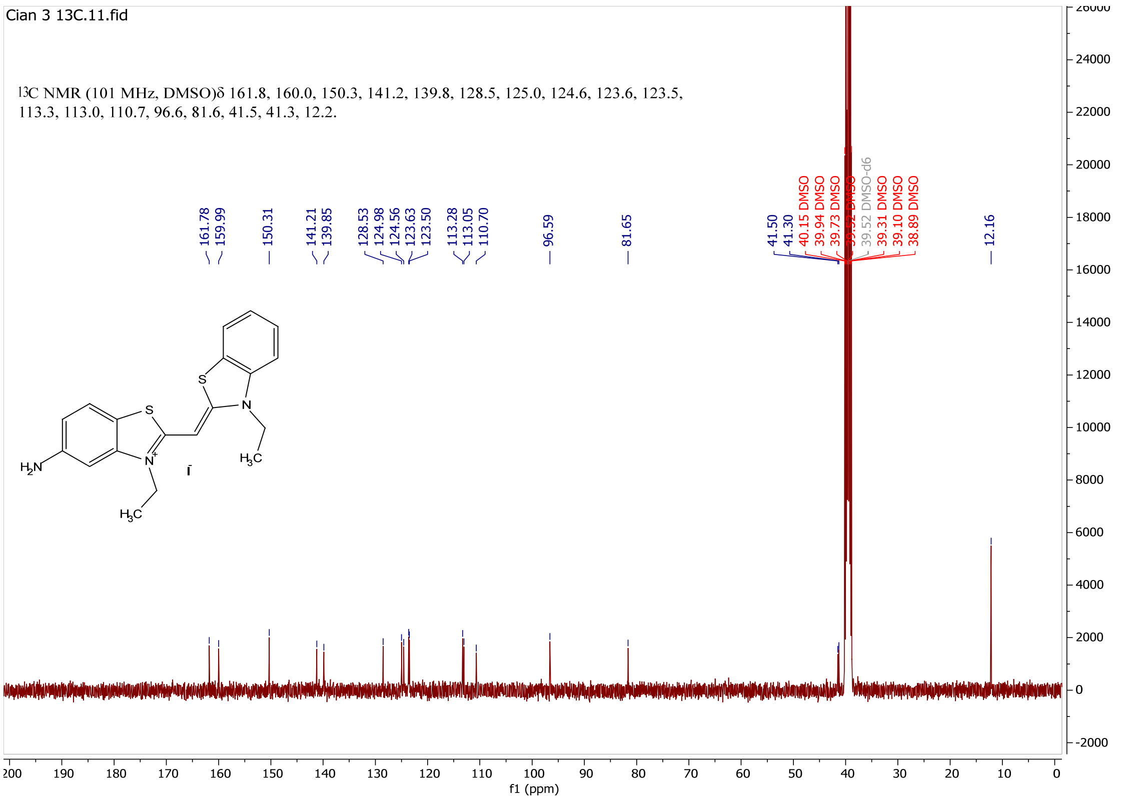

1.10. Figure S10 – ^{1}H and ^{13}C NMR spectra of cyanine dye 8.

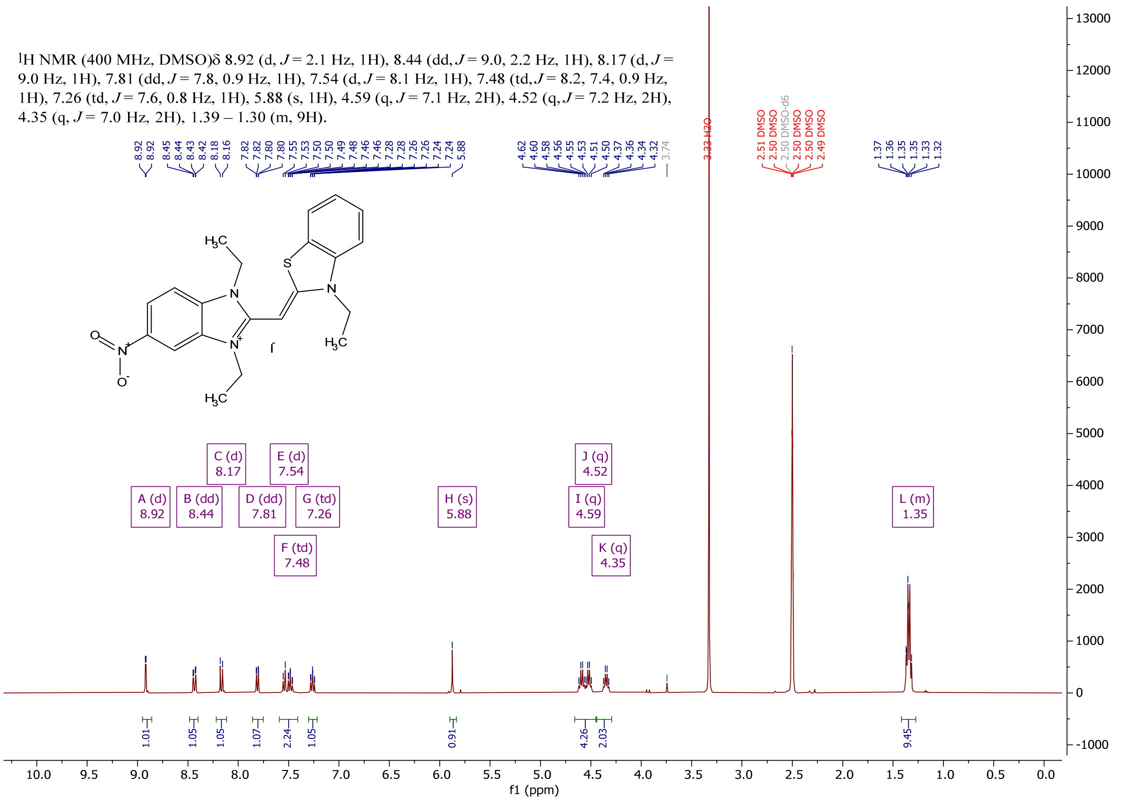

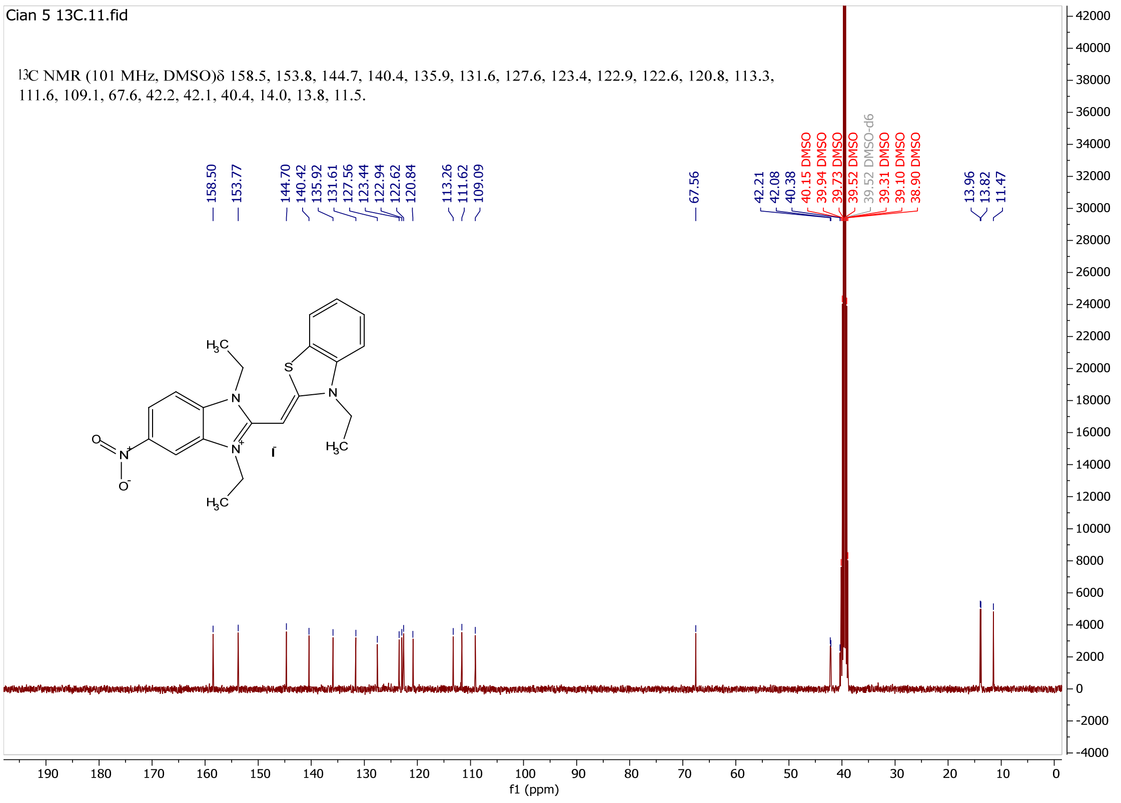

1.11. Figure S11 – ^{1}H and ^{13}C NMR spectra of cyanine dye 9.

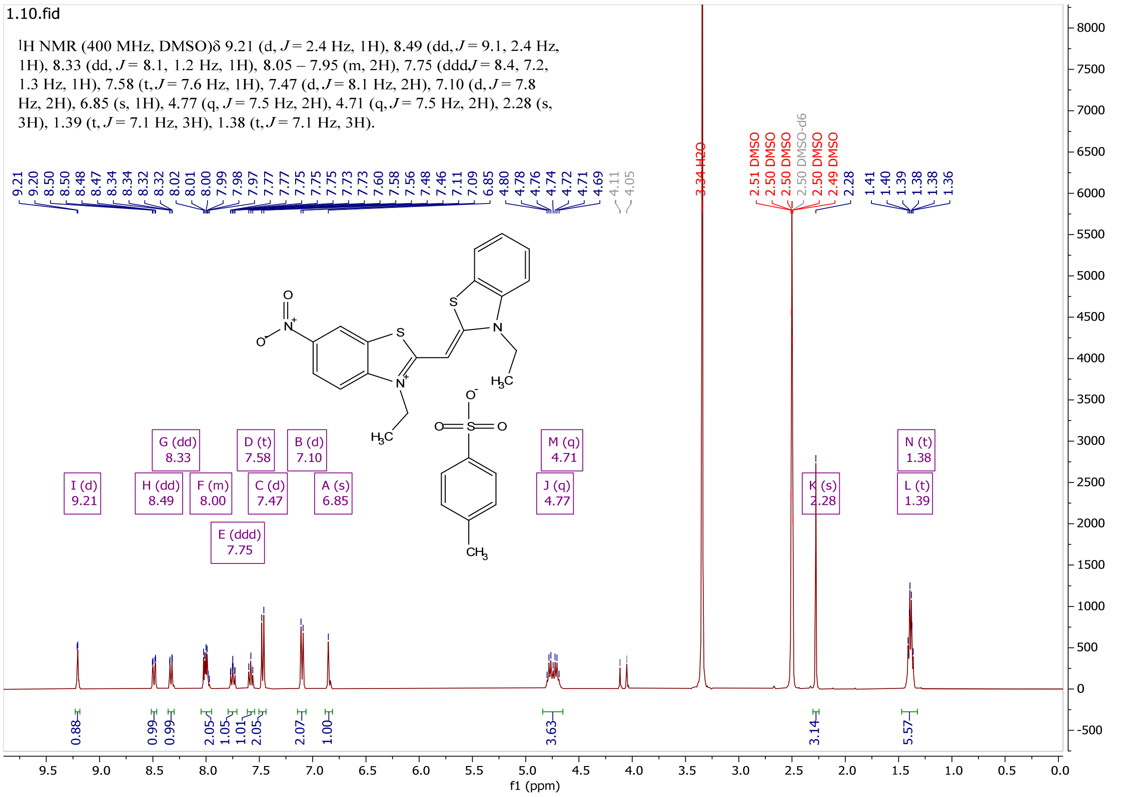

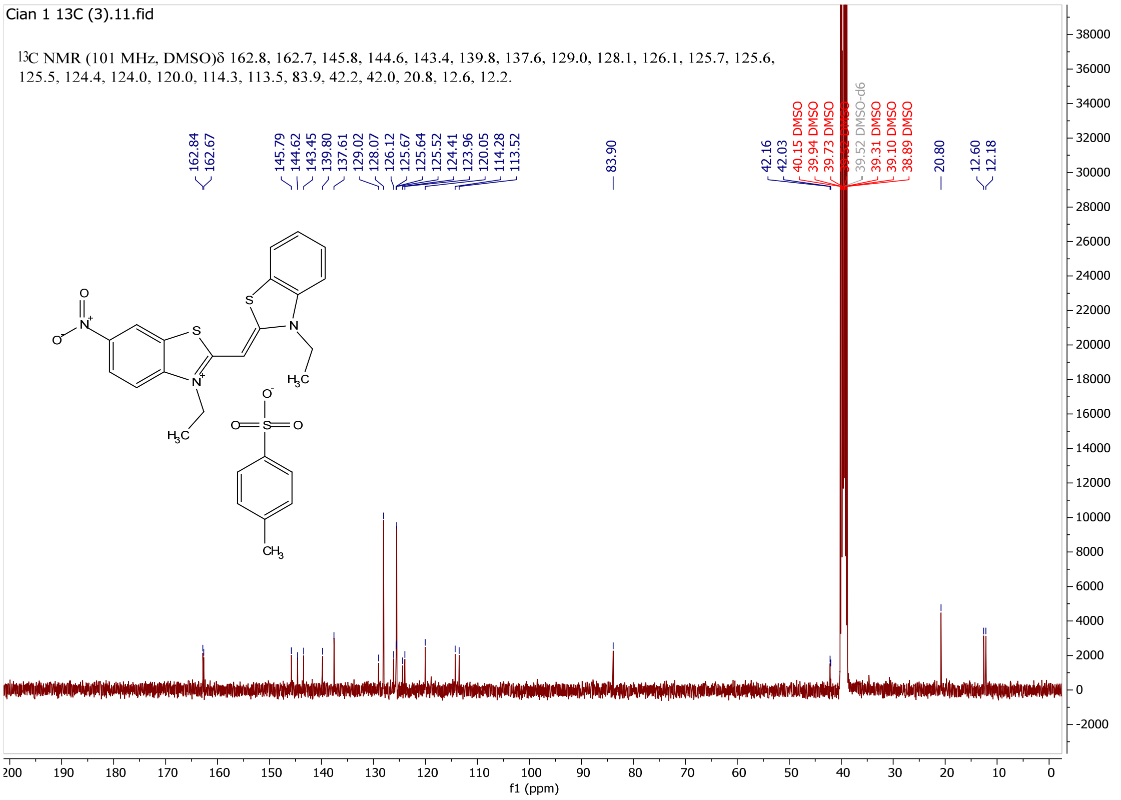

1.12. Figure S12 – ^{1}H and ^{13}C NMR spectra of cyanine dye 10.

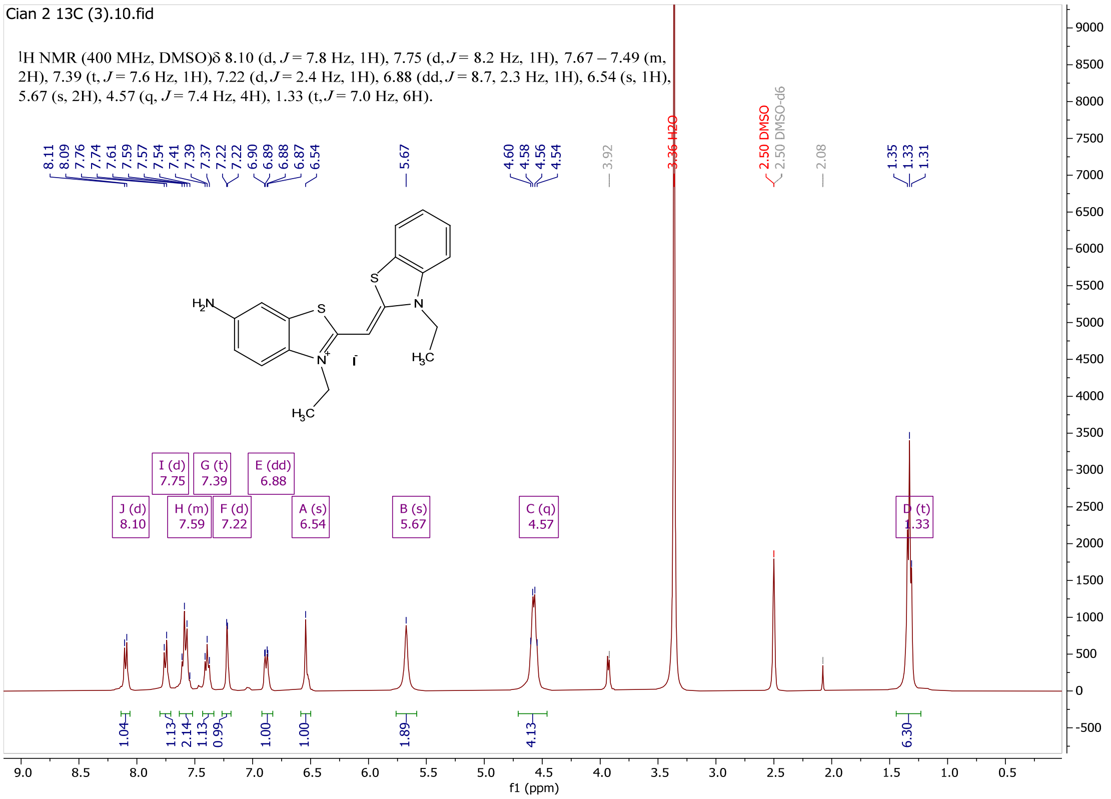

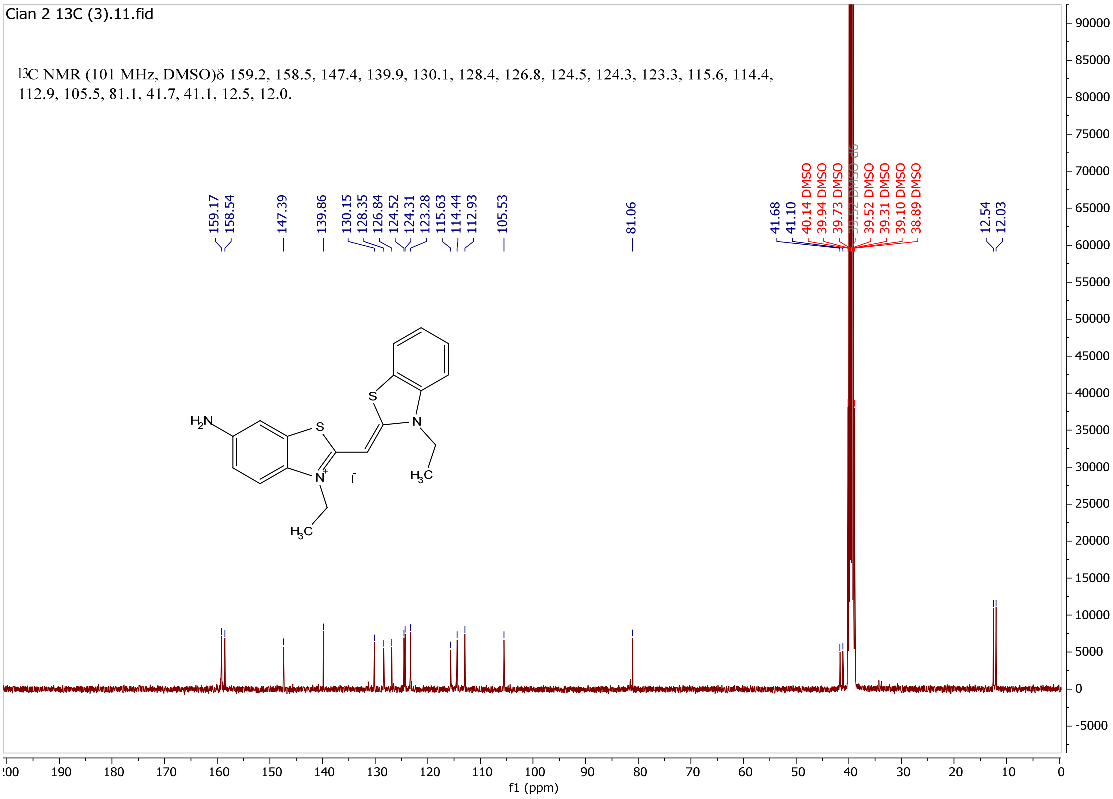

1.13. Figure S13 – ^{1}H and ^{13}C NMR spectra of cyanine dye 11.

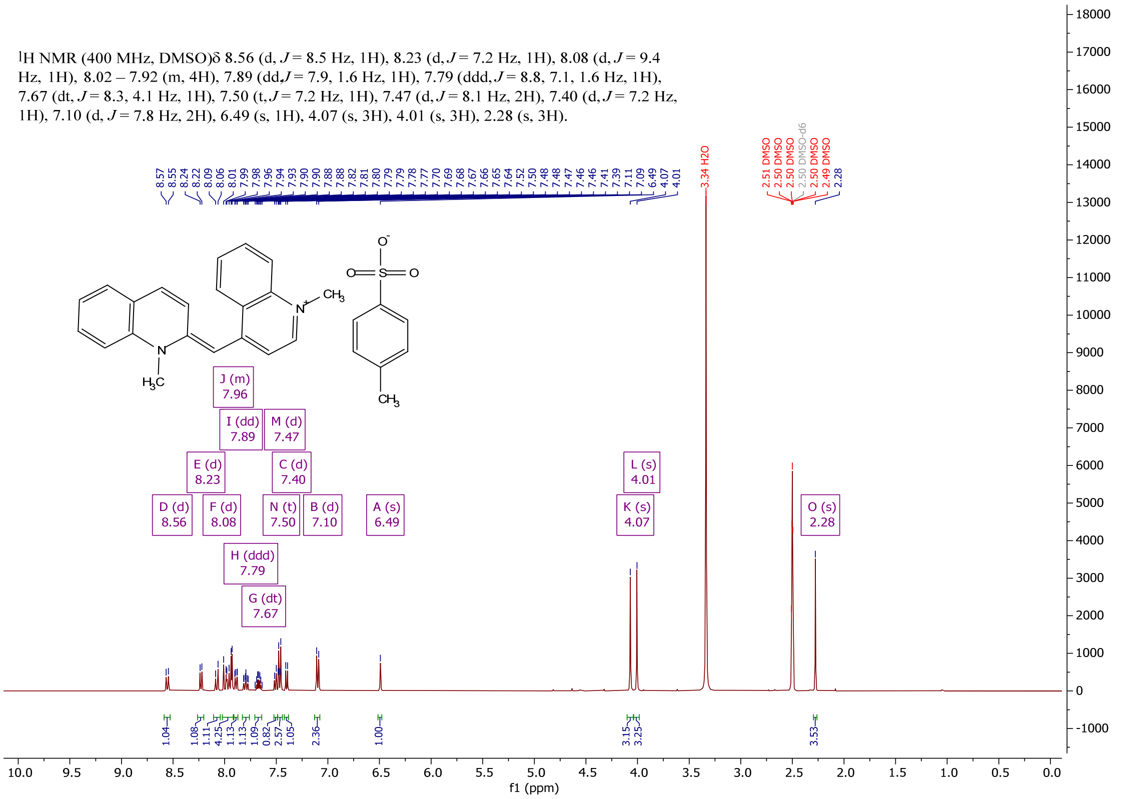

1H NMR (400 MHz, DMSO) δ 8.56 (d, J = 8.5 Hz, 1H), 8.23 (d, J = 7.2 Hz, 1H), 8.08 (d, J = 9.4 Hz, 1H), 8.02 – 7.92 (m, 4H), 7.89 (dd, J = 7.9, 1.6 Hz, 1H), 7.79 (ddd, J = 8.8, 7.1, 1.6 Hz, 1H), 7.67 (dt, J = 8.3, 4.1 Hz, 1H), 7.50 (t, J = 7.2 Hz, 1H), 7.47 (d, J = 8.1 Hz, 2H), 7.40 (d, J = 7.2 Hz, 1H), 7.10 (d, J = 7.8 Hz, 2H), 6.49 (s, 1H), 4.07 (s, 3H), 4.01 (s, 3H), 2.28 (s, 3H).
3.34 H2O
f1 (ppm)

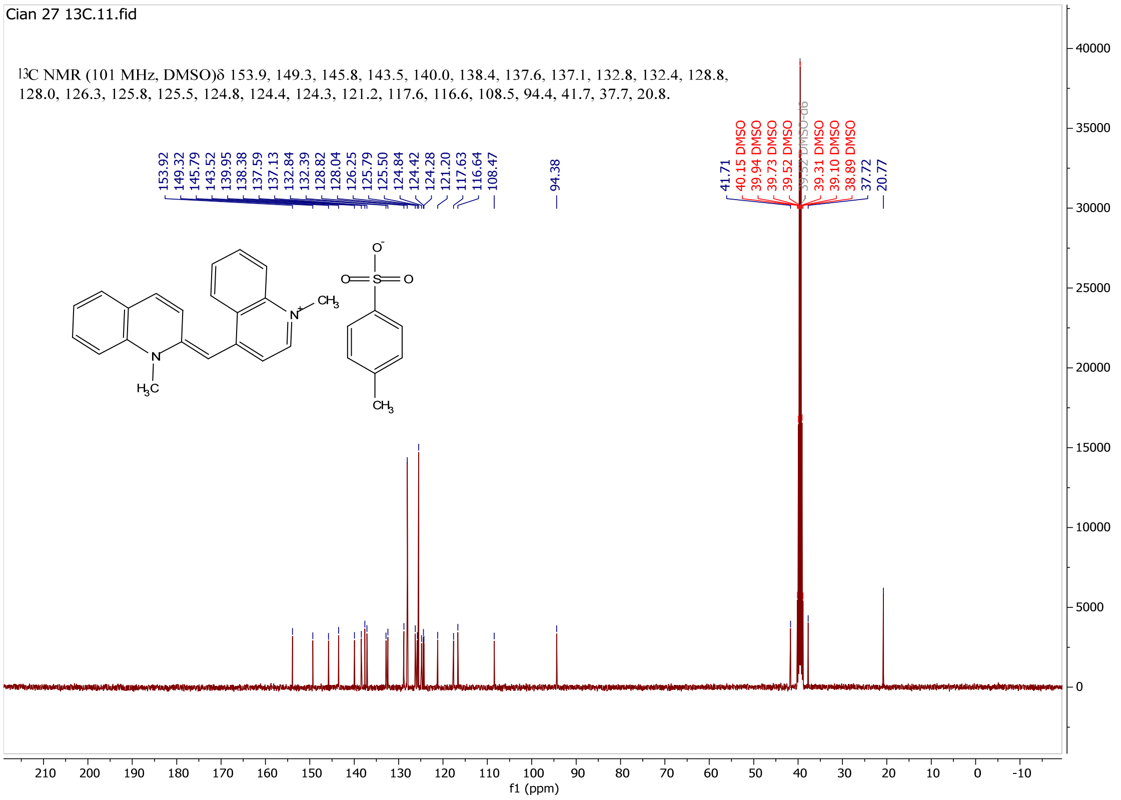

Cian 27 13C.11.fid
13C NMR (101 MHz, DMSO) δ 153.9, 149.3, 145.8, 143.5, 140.0, 138.4, 137.6, 137.1, 132.8, 132.4, 128.8, 128.0, 126.3, 125.8, 125.5, 124.8, 124.4, 124.3, 121.2, 117.6, 116.6, 108.5, 94.4, 41.7, 37.7, 20.8.
f1 (ppm)

1.14. Figure S14 – ^{1}H and ^{13}C NMR spectra of cyanine dye 12.

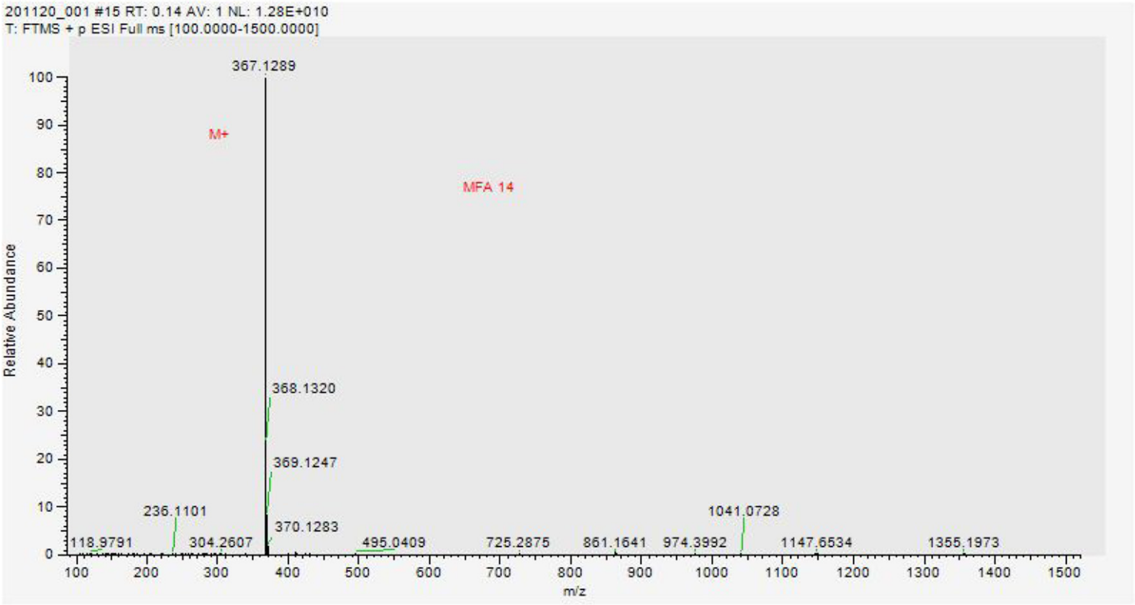

1.15. Figure S15 –HRMS spectrum of cyanine dye 1.

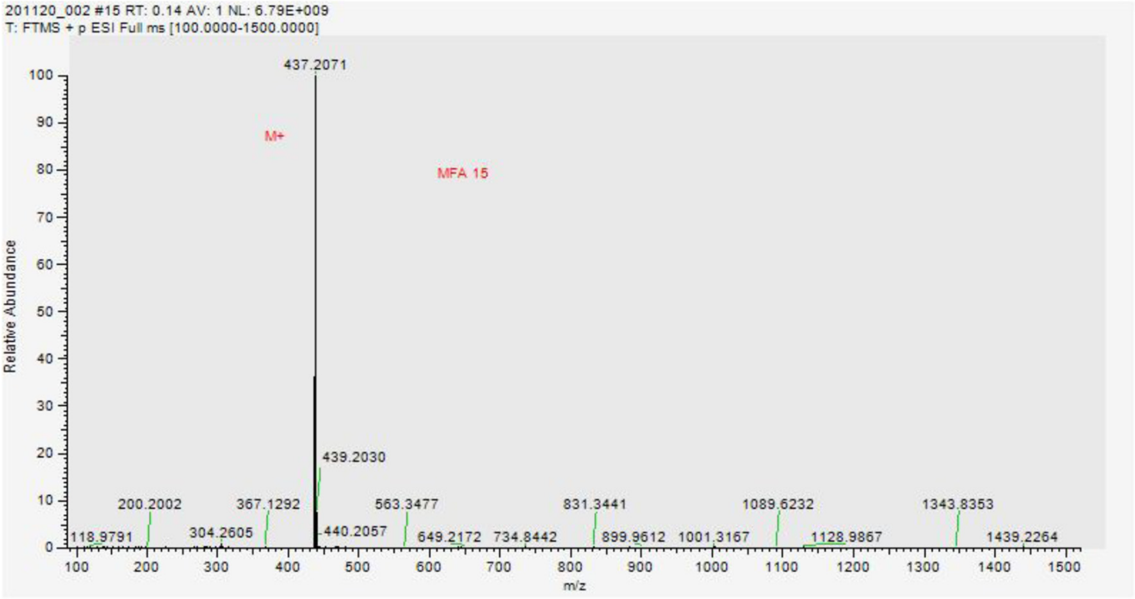

1.16. Figure S16 –HRMS spectrum of cyanine dye 2.

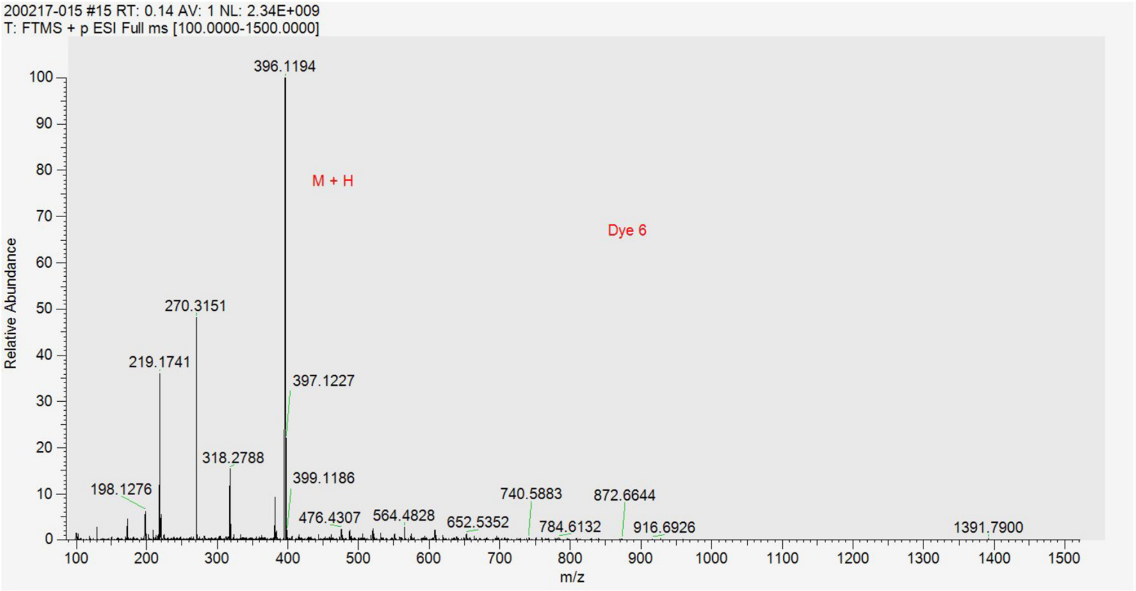

1.17. Figure S17 –HRMS spectrum of cyanine dye 7.

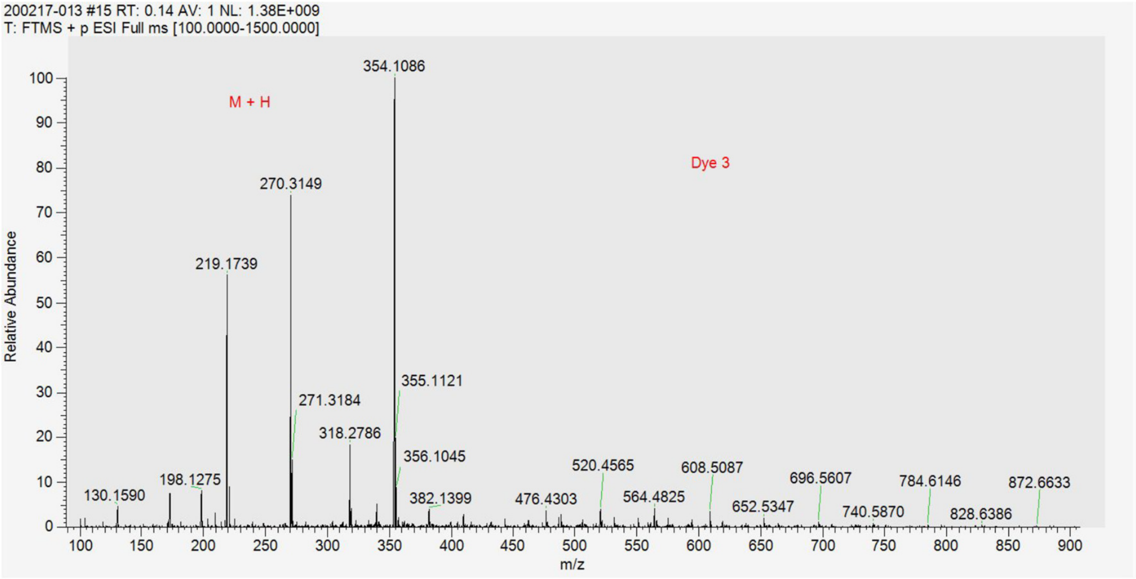

1.18. Figure S18 –HRMS spectrum of cyanine dye 8.

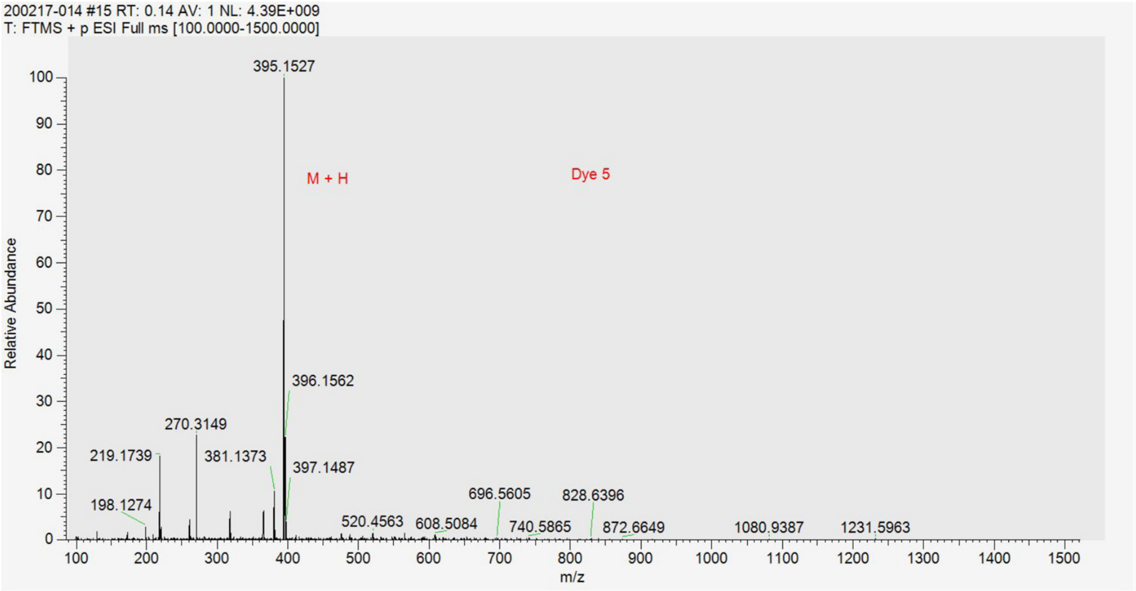

1.19. Figure S19 –HRMS spectrum of cyanine dye 9.

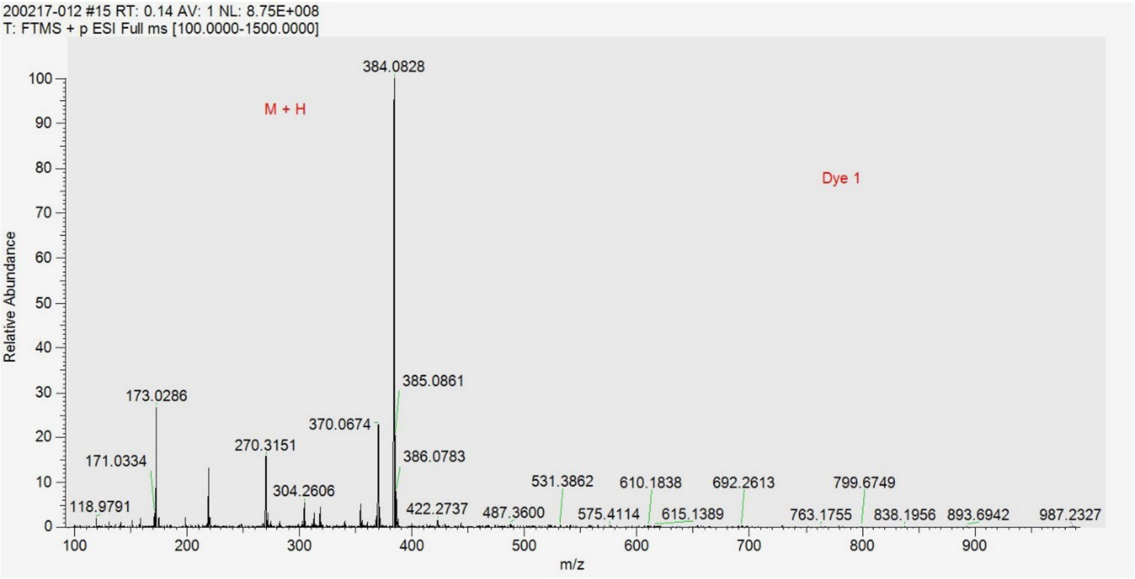

1.20. Figure S20 –HRMS spectrum of cyanine dye 10.
